# A CO_2_ sensing module modulates β-1,3-glucan exposure in *Candida albicans*

**DOI:** 10.1128/mbio.01898-23

**Published:** 2024-01-23

**Authors:** Gabriela M. Avelar, Arnab Pradhan, Qinxi Ma, Emer Hickey, Ian Leaves, Corin Liddle, Alejandra V. Rodriguez Rondon, Ann-Kristin Kaune, Sophie Shaw, Corinne Maufrais, Natacha Sertour, Judith M. Bain, Daniel E. Larcombe, Leandro J. de Assis, Mihai G. Netea, Carol A. Munro, Delma S. Childers, Lars P. Erwig, Gordon D. Brown, Neil A. R. Gow, Marie-Elisabeth Bougnoux, Christophe d'Enfert, Alistair J. P. Brown

**Affiliations:** 1 Institute of Medical Sciences, University of Aberdeen, Foresterhill, Aberdeen, United Kingdom; 2 Medical Research Council Centre for Medical Mycology, School of Biosciences, University of Exeter, Exeter, United Kingdom; 3 Bioimaging Unit, University of Exeter, Exeter, United Kingdom; 4 Centre for Genome Enabled Biology and Medicine, University of Aberdeen, Aberdeen, United Kingdom; 5 Institut Pasteur, Université Paris Cité, INRAe USC2019, Unité Biologie et Pathogénicité Fongiques, Paris, France; 6 Institut Pasteur, Université Paris Cité, Bioinformatics and Biostatistics Hub, Paris, France; 7 Department of Internal Medicine and Radboud Center for Infectious Diseases, Radboud University Medical Center, Nijmegen, the Netherlands; 8 Department for Immunology & Metabolism, Life and Medical Sciences Institute (LIMES), University of Bonn, Bonn, Germany; 9 Johnson-Johnson Innovation, EMEA Innovation Centre, London, United Kingdom; 10 Unité de Parasitologie-Mycologie, Service de Microbiologie Clinique, Hôpital Necker-Enfants-Malades, Assistance Publique des Hôpitaux de Paris (APHP), Paris, France; 11 Université Paris Cité, Paris, France; Duke University Hospital, Durham, USA

**Keywords:** *Candida albicans*, pathogen-associated molecular patterns, β-glucan masking, carbonic anhydrase, *NCE103*, immune evasion

## Abstract

Microbial species capable of co-existing with healthy individuals, such as the commensal fungus *Candida albicans,* exploit multifarious strategies to evade our immune defenses. These strategies include the masking of immunoinflammatory pathogen-associated molecular patterns (PAMPs) at their cell surface. We reported previously that *C. albicans* actively reduces the exposure of the proinflammatory PAMP, β-1,3-glucan, at its cell surface in response to host-related signals such as lactate and hypoxia. Here, we show that clinical isolates of *C. albicans* display phenotypic variability with respect to their lactate- and hypoxia-induced β-1,3-glucan masking. We have exploited this variability to identify responsive and non-responsive clinical isolates. We then performed RNA sequencing on these isolates to reveal genes whose expression patterns suggested potential association with lactate- or hypoxia-induced β-1,3-glucan masking. The deletion of two such genes attenuated masking: *PHO84* and *NCE103*. We examined *NCE103*-related signaling further because *NCE103* has been shown previously to encode carbonic anhydrase, which promotes adenylyl cyclase-protein kinase A (PKA) signaling at low CO_2_ levels. We show that while CO_2_ does not trigger β-1,3-glucan masking in *C. albicans*, the Sch9-Rca1-Nce103 signaling module strongly influences β-1,3-glucan exposure in response to hypoxia and lactate. In addition to identifying a new regulatory module that controls PAMP exposure in *C. albicans,* our data imply that this module is important for PKA signaling in response to environmental inputs other than CO_2_.

**IMPORTANCE:**

Our innate immune defenses have evolved to protect us against microbial infection in part via receptor-mediated detection of “pathogen-associated molecular patterns” (PAMPs) expressed by invading microbes, which then triggers their immune clearance. Despite this surveillance, many microbial species are able to colonize healthy, immune-competent individuals, without causing infection. To do so, these microbes must evade immunity. The commensal fungus *Candida albicans* exploits a variety of strategies to evade immunity, one of which involves reducing the exposure of a proinflammatory PAMP (β-1,3-glucan) at its cell surface. Most of the β-1,3-glucan is located in the inner layer of the *C. albicans* cell wall, hidden by an outer layer of mannan fibrils. Nevertheless, some β-1,3-glucan can become exposed at the fungal cell surface. However, in response to certain specific host signals, such as lactate or hypoxia, *C. albican*s activates an anticipatory protective response that decreases β-1,3-glucan exposure, thereby reducing the susceptibility of the fungus to impending innate immune attack. Here, we exploited the natural phenotypic variability of *C. albicans* clinical isolates to identify strains that do not display the response to β-1,3-glucan masking signals observed for the reference isolate, SC5314. Then, using genome-wide transcriptional profiling, we compared these non-responsive isolates with responsive controls to identify genes potentially involved in β-1,3-glucan masking. Mutational analysis of these genes revealed that a sensing module that was previously associated with CO_2_ sensing also modulates β-1,3-glucan exposure in response to hypoxia and lactate in this major fungal pathogen of humans.

## INTRODUCTION

Relatively few of the millions of fungal species that inhabit our planet enjoy symbiotic relationships with humans (1, 2). However, those species that can colonize humans display great phenotypic diversity, having emerged in different phylogenetic branches of the fungal kingdom and having been exposed to different evolutionary pressures (3, 4). Nevertheless, those fungi that are able to co-exist with healthy individuals, via parasitic, commensal, or mutualistic relationships, must have evolved strategies to evade or overcome the local immune defenses of their host (5–8). In principle, such strategies could include constitutive bet hedging through the generation of phenotypically heterogeneous populations that include subsets of cells with a higher probability of surviving an impending challenge (9, 10). They might also involve the induction of anticipatory responses, whereby the fungus has evolved to exploit one type of environmental input to activate a response to a second, impending challenge (7, 11–13). This type of anticipatory response, which involves temporally related environmental inputs, is thought to have led to the development of core environmental responses in fungi (7, 12, 14) and has been termed “adaptive prediction” (11).

Constitutive bet hedging and inducible anticipatory responses play important roles in fungal immune evasion. For example, the human commensal fungus *Candida albicans* exploits both strategies to avoid the recognition, by innate immune cells, of the essential but immunoinflammatory pathogen-associated molecular pattern (PAMP) β-1,3-glucan. β-1,3-Glucan is an essential component of the *C. albicans* cell wall, comprising about 75% of cell wall biomass (15, 16). Most of the β-1,3-glucan lies in the inner layer of the cell wall, buried below the outer layer of mannan fibrils (16), but some β-1,3-glucan can become exposed at the *C. albicans* cell surface at septal junctions, bud scars, and at punctate foci on the lateral cell wall (17). This exposed β-1,3-glucan becomes visible to host pattern recognition receptors (PRRs) such as the C-type lectin receptor dectin-1 (CLEC7A), the nucleotide-oligomerization domain-like receptor NLRP3, and complement receptor 3 (18–22). The recognition of β-1,3-glucan by these PRRs, and by dectin-1 in particular, plays a major role in antifungal immunity (23–29), triggering a range of responses that promote fungal killing and clearance from the infection site. These responses include phagocytosis, the formation of neutrophil extracellular traps, and cytokine release with the ensuing recruitment of innate immune cells and induction of adaptive immune responses (21, 22, 30, 31). However, *C. albicans* has evolved a variety of mechanisms to counter β-1,3-glucan-mediated immune recognition. First, even under steady-state conditions, *C. albicans* cell populations display a high degree of phenotypic variability with respect to their levels of β-1,3-glucan exposure, with subsets of cells revealing minimal β-1,3-glucan (32–34). Second, daughter cells are less visible to innate immune cells, displaying relatively low levels of β-1,3-glucan exposure compared to their mothers (17), probably through asymmetric expression of the Eng1 endoglucanase during cytokinesis (35–37). Third, *C. albicans* cells actively shave exposed β-1,3-glucan from their cell surface (32–34, 38, 39) by secreting the Xog1 exoglucanase in response to specific host signals that are indicative of impending attack by innate immune cells (40). These signals include exposure to lactate or hypoxia (32, 33), and this β-1,3-glucan shaving attenuates fungal recognition by innate immune cells and subsequent cytokine responses (17, 32–34, 38, 39). Therefore, *C. albicans* combines constitutive bet hedging with anticipatory responses to reduce β-1,3-glucan exposure and evade antifungal immunity.

Some progress has been made in elaborating the molecular mechanisms that drive this anticipatory β-1,3-glucan shaving. For example, lactate-, hypoxia-, iron-limitation- and pH-induced changes in β-1,3-glucan exposure are each activated via evolutionarily conserved signaling pathways that respond to the input signal in question (17, 32–34, 38, 39). For example, lactate-induced β-1,3-glucan masking appears to be dependent on Gpr1, the closest *C. albicans* homolog to the mammalian lactate receptor (32), whereas the generation of mitochondrial reactive oxygen species is required for hypoxia-induced β-1,3-glucan masking (33). These input-specific upstream signaling pathways converge on the cyclic AMP (cAMP)-protein kinase A (PKA) pathway (17, 34), which leads to the induction of Xog1 secretion and β-1,3-glucan shaving (17, 40).

Nevertheless, gaps remain in our understanding of the mechanisms that underlie β-1,3-glucan masking and its regulation. Therefore, in this study, we exploited the genetic and phenotypic variability of *C. albicans* clinical isolates to identify new factors involved in these processes. We screened 146 sequenced isolates for their ability to display lactate- and hypoxia-induced β-1,3-glucan masking and then performed RNA sequencing on responsive and non-responsive isolates to identify loci whose (lack of) regulation correlated with a (lack of) β-1,3-glucan masking. Our downstream analysis of nine target loci led to the identification of a new regulatory module that controls β-1,3-glucan masking in *C. albicans*.

## RESULTS

### Clinical isolates of *C. albicans* display variability in their β-glucan masking

**Fig 1:**
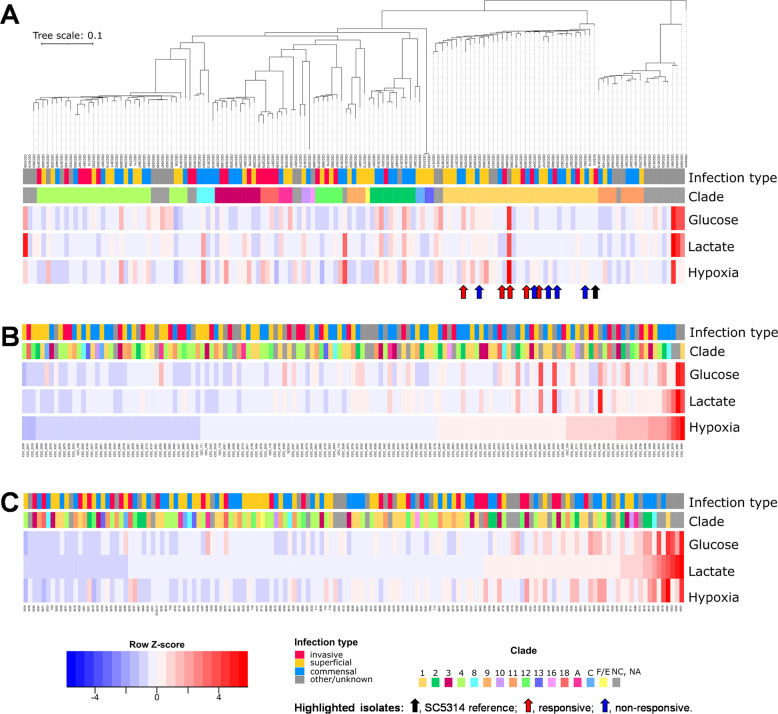
*C. albicans* clinical isolates display variability with respect to their β-1,3-glucan masking phenotypes. The β-1,3-glucan exposure levels for 146 *C*. *albicans* clinical isolates were quantified by Fc-dectin-1 staining and flow cytometry under control conditions (glucose, GYNB) and following exposure to lactate or hypoxia. Row *Z*-scores were calculated based on the mean fluorescence intensities of three biological replicates per condition and visualized in heatmaps: blue, strong β-1,3-glucan masking; white, no significant masking; red, β-1,3-glucan exposure. The cluster to which each clinical isolate belongs is indicated by the color code: NC, no cluster assigned. Also, the nature of the infection from which each isolate was obtained is color coded (see key). The reference *C. albicans* isolate (SC5314, black arrow), the five responsive isolates (CEC3560, CEC3605, CEC3609, CEC4108, CEC4259; red arrows), and five non-responsive isolates selected for further analysis (CEC3534, CEC3544, CEC3621, CEC3636, CEC4035; blue arrows) are highlighted. (**A**) The clinical isolates are clustered with respect to their sequence relatedness. All responsive and non-responsive isolates are from cluster 1. (**B**) The isolates are re-ordered with respect to the strength of their hypoxia-induced β-1,3-glucan masking (MFI hypoxia/MFI glucose control = normoxia). (**C**) The isolates are re-ordered with respect to the strength of their lactate-induced β-1,3-glucan masking (MFI glucose plus with lactate/MFI glucose control).

To explore the extent to which clinical isolates display lactate- and hypoxia-induced β-1,3-glucan masking, we took advantage of a collection of sequenced clinical isolates of *C. albicans* that spans all the major genetic clusters (often referred to as clades) (41). We selected 146 isolates representing the various clusters, including isolates from different types of infection (Table S1). We also included the reference strain SC5314 as a control (Table S2) because this strain provided the platform for previous β-1,3-glucan masking studies (32–34). These isolates were arrayed in 96-well format, and using established approaches (32–34), they were grown in a control glucose-based minimal medium (GYNB) under normoxic conditions and then exposed to lactate or hypoxia for 5 hours before quantifying their β-1,3-glucan exposure levels. To achieve this, cells were harvested during exponential growth under each of these conditions, fixed, stained with Fc-dectin-1, and subjected to flow cytometry. Three independent measurements were taken for each isolate/condition. The median fluorescence indices (MFIs) of these cells were compared to those for control cells that were incubated in GYNB without lactate under normoxic conditions to reveal the degree of lactate- and hypoxia-induced β-1,3-glucan masking for each isolate. This screen revealed that *C. albicans* isolates display a high degree of variability in their β-1,3-glucan masking capacity under these experimental conditions (Fig. 1). Some isolates, like SC5314, displayed efficient lactate- and/or hypoxia-induced β-1,3-glucan masking, whereas others displayed modest β-1,3-glucan masking, and others even showed elevated β-1,3-glucan exposure at this 5-hour timepoint. The β-1,3-glucan masking phenotype is complex as it is influenced by new cell wall synthesis, cell division, and the shaving of exposed β-1,3-glucan (17, 40). The correlation between the growth of these strains and their β-1,3-glucan exposure was modest (Fig. S1), suggesting that the observed variability in masking partially reflected strain differences in masking dynamics as well as in masking capacity. Hence, the observed strain variability was probably influenced by changes in the signaling pathways that drive cell wall remodeling or β-1,3-glucan masking (33), via alterations in cell wall architecture and the outer mannan layer in particular (42–46), and/or through defects in β-1,3-glucan shaving mechanisms themselves (35, 40). Given this complexity, the lack of an obvious correlation between phylogeny and phenotype for lactate- or hypoxia-induced masking (Fig. 1A) was not unexpected. Furthermore, no correlation was observed between a strain’s β-1,3-glucan masking phenotype and the location from where it was isolated (bloodstream, mucosal surface, or feces) (Fig. 1B and C).

### Transcriptomic changes associated with lactate- or hypoxia-induced β-glucan masking in *C. albicans*

*C. albicans* clinical isolates display a high degree of genetic variation. Indeed, the collection of sequenced isolates from the Institut Pasteur displays, on average, one heterozygous single-nucleotide polymorphism (SNP) every 204 bp and an insertion or deletion (indel) every 944 bp (41). This high degree of genetic variation, together with the complexity of the β-1,3-glucan masking phenotype, precluded the association of specific SNPs or indels with the loss of this phenotype. Therefore, we used RNA sequencing to target loci potentially involved in lactate- or hypoxia-induced β-1,3-glucan masking.

To achieve this, we first selected five clinical isolates that displayed masking in response to lactate and hypoxia (responders: CEC3560, CEC3605, CEC3609, CEC4108, and CEC4259) and five clinical isolates that were defective in lactate- and hypoxia-induced masking (non-responders: CEC3534, CEC3544, CEC3621, CEC3636, and CEC4035). These isolates, like the control strain SC5314, were all selected from cluster 1 to limit inter-strain variability. We performed RNA sequencing on each of these isolates after 1 hour of exposure to lactate or hypoxia and then compared these transcriptomes with those from control normoxic cultures lacking lactate, using data from three independent replicates for each condition (Table S3; https://www.ebi.ac.uk/biostudies/arrayexpress, accession number E-MTAB-10986; data files at www.ebi.ac.uk/ena/browser/home, project PRJEB47705).

**Fig 2:**
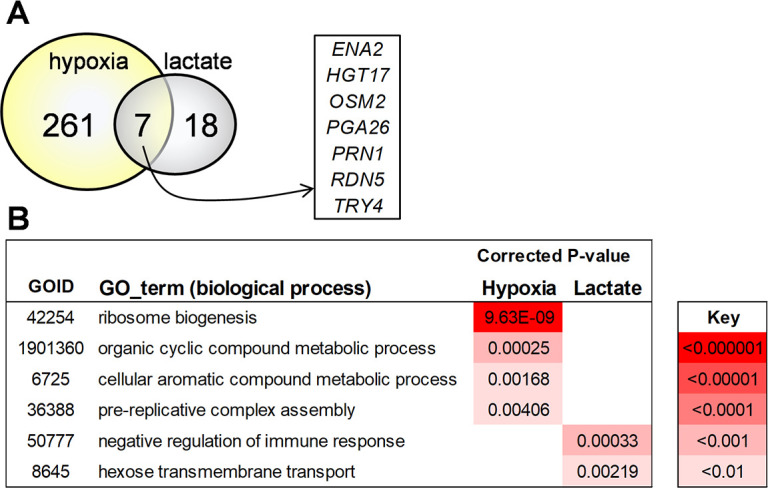
Impact of lactate and hypoxia on the transcriptomes of responsive and non-responsive *C. albicans* clinical isolates. RNA sequencing was performed on five responsive (CEC3560, CEC3605, CEC3609, CEC4108, and CEC4259) and five non-responsive clinical isolates (CEC3534, CEC3544, CEC3621, CEC3636, and CEC4035) following exposure to lactate or hypoxia for 1 hour and compared to the control condition (fresh GYNB for 1 hour). (**A**) Venn diagram showing the numbers of genes that were upregulated (>twofold) in the responsive isolates in response to lactate and hypoxia. Seven genes that were significantly upregulated under both β-1,3-glucan masking conditions are shown. (**B**) Gene ontology terms that were significantly enriched in the gene sets that were upregulated in the responsive isolates in response to lactate and hypoxia. The degree of statistical significance of this enrichment is indicated by the color coding.

The transcriptomic data for each of the five responder isolates were generated, and then these data were used to identify genes that were upregulated in each of these strains in response to the different β-1,3-glucan masking conditions. We identified 268 genes that were significantly upregulated more than twofold in response to hypoxia and 25 genes that were upregulated following lactate exposure (Fig. 2A). The set of hypoxia-induced genes displayed significant enrichment in gene ontology (GO) terms relating to ribosome biogenesis, DNA replication, and metabolism, whereas the lactate-induced genes were enriched in hexose transporters and the negative regulation of immune responses (Fig. 2B; Tables S3 and S4). Seven genes were upregulated under both conditions in the five responder isolates (Fig. 2). These genes encode proteins involved in a variety of processes, including metabolism (*HGT17, OSM2*), ribosome biogenesis (*RDN5*), transcription (*PRN1, TRY4*), cell wall biogenesis (*PGA26*), and stress resistance (*ENA2*).

**Fig 3:**
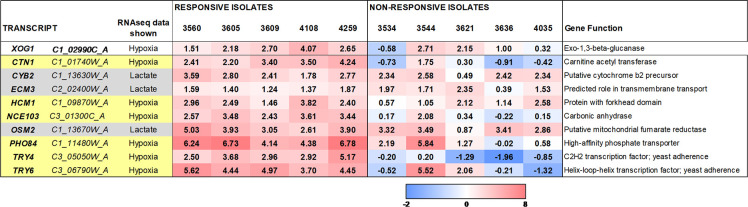
*C. albicans* genes targeted for further analysis based on RNA sequencing. Nine *C. albicans* genes were targeted for analysis based on their transcriptional responses in the responsive and non-responsive clinical isolates to either lactate or hypoxia: lactate, pale gray; hypoxia, pale yellow. The regulation of their transcripts in response to lactate or hypoxia in each of the responsive and non-responsive isolates is shown, along with their (putative) functions.

We then examined the transcriptomic data for the five non-responder isolates. Overall, the non-responders displayed similar transcriptomic changes to the responders (Tables S3 and S4). This was not surprising because, given the genetic diversity of these isolates (41), each of the non-responders is likely to carry different β-1,3-glucan masking defects. Therefore, to select loci that might be involved in β-1,3-glucan masking, we first rank ordered genes based on the strength of their upregulation in response to lactate or hypoxia. We focused on genes that were upregulated at least twofold in all five responder strains. Using this rank-ordered list of upregulated genes, we then selected genes whose upregulation was lost or dramatically reduced in at least one of the non-responding strains. *XOG1,* which encodes the major secreted exoglucanase that promotes β-1,3-glucan shaving (40, 47), was upregulated in response to hypoxia in all five responding isolates, and this upregulation was lost in three of the five non-responding isolates (Fig. 3). Although Xog1 levels appear to be post-transcriptionally modulated (17), these differences in *XOG1* regulation between isolates did support the rationale underpinning our approach.

On this basis, nine *C. albicans* genes were targeted for analysis (Fig. 3). Three of these were selected based on their responses to lactate (*CYB2, ECM3, OSM2*) and the other six based on their responses to hypoxia (*CTN1, HCM1, NCE103, PHO84, TRY4, TRY6*) (Fig. 3). Some of these genes are predicted to encode transporters or metabolic functions (*CTN1, CYB2, ECM3, NCE103, OSM2, PHO84*), some are associated with mitochondrial functionality (*CTN1, CYB2, HCM1, OSM2*), which has been associated with hypoxia-induced β-1,3-glucan masking (33), and others encode putative transcriptional regulators (*HCM1, TRY4, TRY6*).

### Impact of *CTN1, CYB2, ECM3, HCM1, NCE103, OSM2, PHO84, TRY4,* and *TRY6* upon lactate- and hypoxia-induced β-glucan masking in *C. albicans*

To test whether any of the nine selected target genes play a role in β-1,3-glucan masking, we generated two independent homozygous null mutants for each target gene in *C. albicans* strain SC5314 using CRISPR-Cas9 technology, and their genotypes were confirmed by diagnostic PCR (Fig. S2). First, the sensitivities of these null mutants to cell wall and environmental stresses were compared to those of the control parental strain. In all cases, each pair of independent null mutants behaved in a similar manner. The *hcm1* mutants grew slowly compared to the parental wild-type strain SC5314, thereby recapitulating this reported phenotype for *hcm1* cells (48). The *hcm1* mutants were also sensitive to amino acid starvation (10 mM 3-aminotriazole) and thermosensitive at temperatures above 42°C (Fig. S3). The *ctn1* mutants were unable to grow on non-fermentable carbon sources, and the *nce103* mutants were auxotrophic for CO_2_, as reported previously (49, 50). Therefore, stress sensitivities of the *ctn1* mutants were assayed during growth on glucose, and *nce103* stress phenotypes were examined under high CO_2_. None of the mutants displayed sensitivity to antifungal drugs (0.65 µg/mL fluconazole; 0.032 µg/mL caspofungin; 5 µg/mL Ambisome), cell wall stresses (1 mg/mL caffeine; 60 µg/mL calcofluor white; 0.3 mg/mL Congo red), osmotic stress (1 M NaCl; 0.6 M KCl), oxidative stress (5 mM H_2_O_2_; 0.3 mM menadione), reductive stress (25 mM dithiothreitol), weak acid stress (20 mM acetic acid, pH 3), copper (5 mM CuSO_4_), iron (100 µM FeCl_3_), or amino acid starvation (10 mM 3-aminotriazole) in our hands (Table S5).

**Fig 4:**
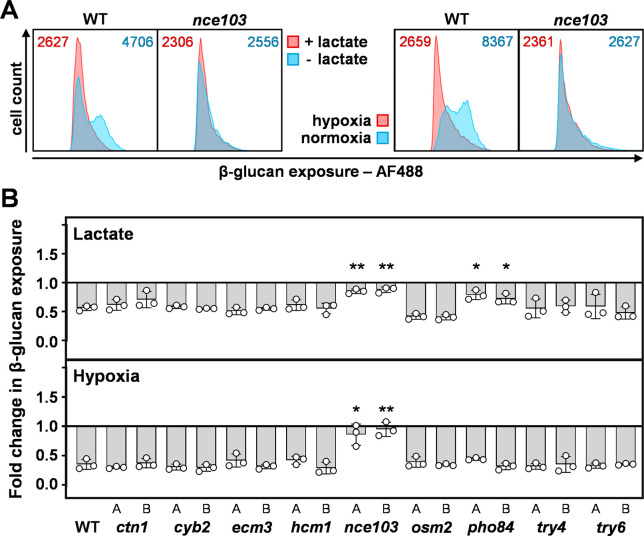
Impact of the target loci upon lactate- and hypoxia-induced β-1,3-glucan masking in *C. albicans*. Each target locus was deleted in *C. albicans* SC5134 (WT), and the degree of β-1,3-glucan masking quantified each mutant in response to lactate and hypoxia via Fc-dectin-1 staining and flow cytometry. (**A**) Representative cytometry plots from three independent experiments showing the β-1,3-glucan exposure for *nce103* cells and their wild type control (SC5134). The left-hand panels show responses to lactate, and the right-hand panels show the responses to hypoxia: blue, control, no masking stimulus; pink, plus masking stimulus. The corresponding MFIs are shown at the top of each panel. (**B**) Two independent homozygous null mutants (**A, B**) were analyzed for each target gene. Fold changes in β-1,3-glucan exposure were calculated by dividing the MFI for lactate- or hypoxia-exposed cells by the MFI for the corresponding normoxic GYNB control (Materials and Methods). Means and standard deviations from three independent replicate experiments are shown, and the data were analyzed using ANOVA with Tukey’s multiple comparison test: *, *P* < 0.05; **, *P* < 0.01.

We then quantified the ability of each *C. albicans* mutant to activate β-1,3-glucan masking in response to lactate or hypoxia. Cells were fixed, stained with Fc-dectin-1 and AF488-linked secondary antibody, and their fluorescence quantified by flow cytometry. The parental SC5314 cells displayed significant population heterogeneity with respect to their β-1,3-glucan exposure, and robust β-1,3-glucan masking following exposure to lactate or hypoxia (Fig. 4A), as described previously (32, 33). Interestingly both *nce103* and *pho84* mutants displayed significantly attenuated masking in response to lactate, partly due to their low levels of β-1,3-glucan exposure under the control condition (Fig. 4B; Table S6). The pair of *nce103* mutants also displayed a significant defect in response to hypoxia (Fig. 4B). None of the other mutants displayed aberrant β-1,3-glucan masking, suggesting that the correlations in gene regulation that we had observed for these genes (Fig. 3) did not reflect causative effects on β-1,3-glucan masking.

*PHO84* (C1_11,480W_A) encodes a high-affinity phosphate transporter, the inactivation of which increases the sensitivity to neutrophil killing and attenuates the virulence of *C. albicans,* through increased sensitivity to oxidative stress (40 mM H_2_O_2_) (51). We did not pursue *PHO84* further as *pho84* cells only displayed a defect in lactate-induced masking. Instead, we chose to examine *NCE103* because *nce103* cells were defective in both lactate- and hypoxia-induced masking and because of its links to cAMP-PKA signaling (below), a pathway that has been implicated in β-1,3-glucan masking (33, 34).

*NCE103* (C3_01,300C_A) encodes carbonic anhydrase, which accelerates the conversion of dissolved CO_2_ to bicarbonate (50). *NCE103* enhances the growth of *C. albicans* in air (which contains about 0.04% CO_2_) and is required for the virulence of this fungus in host niches with limited CO_2_ (50). *Nce103* cells grow under high CO_2_ (5%) through the chemical formation of bicarbonate from CO_2_ (Fig. S3A) (50). Bicarbonate directly stimulates the activity of adenylyl cyclase, thereby enhancing cAMP-PKA signaling in *C. albicans* (50). PKA signaling is required for both lactate- and hypoxia-induced β-1,3-glucan masking (33), and high CO_2_ levels are often associated with hypoxic microenvironments *in vivo* (52). Consequently, our finding that Nce103 is required for both lactate- and hypoxia-induced changes in β-1,3-glucan exposure was intriguing. Therefore, we explored the roles of CO_2_ and Nce103 signaling in β-1,3-glucan masking in *C. albicans*.

### Impact of CO_2_ upon β-1,3-glucan masking in *C. albicans*

**Fig 5:**
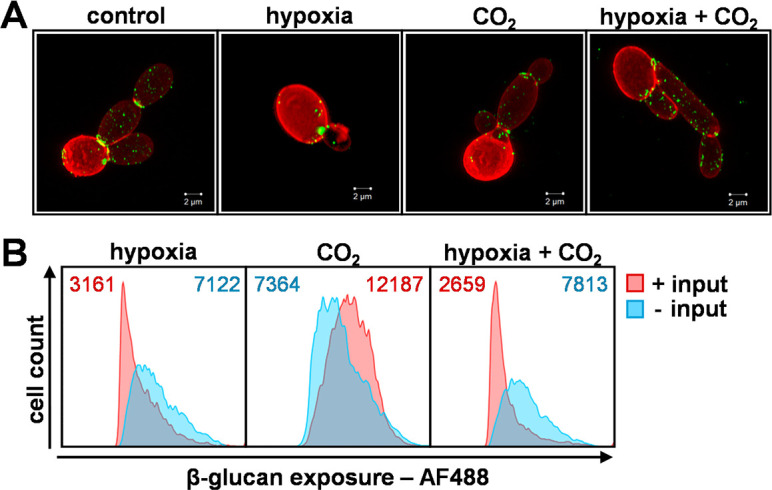
Effect of CO_2_ upon β-1,3-glucan exposure in *C. albicans*. *C. albicans* SC5134 cells were exposed for 5 hours to 5% CO_2_, hypoxia, or a combination of the two, and compared with control cells grown in normoxic GYNB without CO_2_. (**A**) These cell populations were then stained with Fc-dectin-1 and subjected to flow cytometry to quantify their levels of β-1,3-glucan exposure. The plots shown are representative of three independent experiments, with the corresponding MFIs presented at the top of each panel: blue, control, no input; pink, plus input. (**B**) In parallel, these cell populations were double stained with Fc-dectin-1 (exposed β-1,3-glucan, AF488, green) and ConA (mannan, AF647, red) and then examined by high-resolution fluorescence confocal microscopy. The images are representative of three independent experiments; scale bar = 2 µm.

To test the impact of CO_2_ upon β-1,3-glucan masking, *C. albicans* SC5314 cells were grown in glucose minimal medium and exposed to 5% CO_2_, hypoxia, or hypoxia plus 5% CO_2_. The levels of β-1,3-glucan exposure were then compared to untreated control cells by high-resolution fluorescence microscopy of Fc-dectin-1-stained cells (Fig. 5A) and β-1,3-glucan exposure quantified by flow cytometry (Fig. 5B). Once again, hypoxia was shown to induce robust β-1,3-glucan masking in wild-type cells. However, exposure to 5% CO_2_ did not (Fig. 5). Meanwhile, the combination of hypoxia and 5% CO_2_ led to β-1,3-glucan masking (Fig. 5), indicating that, in *C. albicans,* the hypoxic signal is dominant over the 5% CO_2_ signal, at least with respect to the β-1,3-glucan masking phenotype.

### Sch9-Rca1-Nce103 signaling regulates the effects of hypoxia and CO_2_ upon β-1,3-glucan masking in *C. albicans*

Carbonic anhydrase (Nce103) plays a critical role in CO_2_ signaling in *C. albicans* through homeostatic control of the intracellular levels of bicarbonate, which interacts with and activates adenylyl cyclase (50). The expression of Nce103 is regulated by the bZIP transcription factor Rca1*,* which binds directly at the *NCE103* locus to induce its transcription at low CO_2_ levels (53). In addition, Nce103 levels are downregulated at high CO_2_ levels. This downregulation is mediated by the protein kinase Sch9, which phosphorylates and inhibits Rca1 in response to high CO_2_, leading to reduced *NCE103* transcription, a mechanism that is conserved in *Saccharomyces cerevisiae* and *Candida glabrata* (54). Therefore, we tested whether this Sch9-Rca1-Nce103 module controls β-1,3-glucan exposure in *C. albicans*.

**Fig 6:**
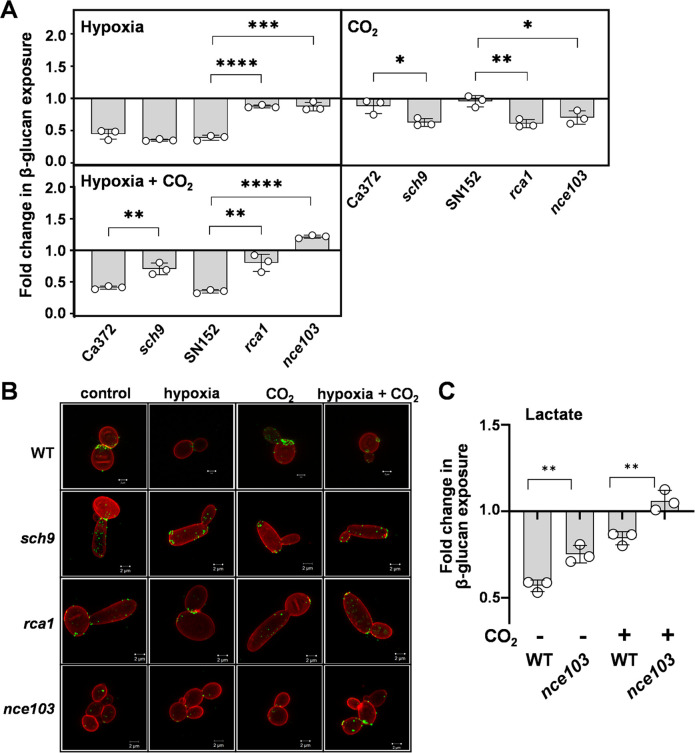
Impact of Nce103, Rca1, and Sch9 upon the changes in β-1,3-glucan exposure mediated by hypoxia and CO_2_. *C. albicans* cells were grown in GYNB at 30°C and exposed to hypoxia, 5% CO_2_, or a combination of these two inputs. (**A**) For each strain, the fold changes in β-1,3-glucan exposure were quantified by Fc-dectin-1 staining and flow cytometry, relative to the same strain grown in normoxic GYNB without CO_2_. *C. albicans* Ca372 (CAI4 + CIp10) is the wild-type control for *sch9* (CAS4), and SN152 is the wild-type control for *rca1* (rca1ΔY) (Table S2). Means and standard deviations from three independent replicate experiments are shown, and the data were analyzed using ANOVA with Tukey’s multiple comparison test: *, *P* < 0.05; **, *P* < 0.01; ***, *P* < 0.001; ****, *P* < 0.0001. (**B**) Corresponding high-resolution fluorescence confocal images of these cells, double stained with Fc-dectin-1 (exposed β-1,3-glucan, AF488, green) and ConA (mannan, AF647, red). The images are representative of three independent experiments; scale bar = 2 µm. (**C**) Using an analogous approach, the influence of *NCE1* on lactate-induced β-1,3-glucan masking was compared in the presence and absence of 5% CO_2_ using wild-type (SC5314) and *nce103* cells (Table S2). Fold changes in β-1,3-glucan exposure were calculated by dividing the MFI for lactate-exposed cells by the MFI for the corresponding GYNB control (Materials and Methods). Means and standard deviations from three independent replicate experiments are shown, and the data were analyzed using ANOVA with Tukey’s multiple comparison test: **, *P* < 0.01.

We quantified β-1,3-glucan exposure on *C. albicans sch9, rca1,* and *nce103* cells in response to 5% CO_2_, hypoxia, or hypoxia plus 5% CO_2_ in comparison to congenic wild-type controls. As expected (Fig. 4), the inactivation of *NCE103* blocked β-1,3-glucan masking in response to hypoxia and hypoxia plus 5% CO_2_ (Fig. 6). Loss of the transcriptional activator Rca1 yielded a similar phenotype, consistent with the idea that *NCE103* expression is required for hypoxia-related β-1,3-glucan masking. Meanwhile, inactivation of the inhibitory kinase, Sch9, did not affect hypoxia-induced β-1,3-glucan masking, but intriguingly, *sch9* cells displayed a partial attenuation of masking in response to hypoxia plus 5% CO_2_ (Fig. 6). Also, *sch9, rca1,* and *nce103* cells displayed significant β-1,3-glucan masking in response to 5% CO_2_ alone in contrast to their wild-type parents which showed no masking under these conditions (Fig. 6). The basis of this is not clear but might relate to the perturbation of Nce103 protein levels or intracellular bicarbonate concentrations in these strains or, alternatively, the possible regulation of *NCE103* expression by factors other than Sch9 and Rca1 in response to hypoxia.

We also tested whether high CO_2_ levels influence lactate-induced β-1,3-glucan masking. Wild-type cells displayed less lactate-induced masking in the presence of high CO_2_ levels, and the inactivation of *NCE103* attenuated masking in the presence or absence of high CO_2_ levels (Fig. 6C).

Our analyses revealed no sequence variation between the responder and non-responder isolates at *SCH9, RCA1,* or *NCE103,* suggesting that mutations at other loci might influence *NCE103* expression levels. Nevertheless, taken together, our data indicate that the Sch9-Rca1-Nce103 signaling module modulates β-1,3-glucan exposure in response to hypoxia and lactate in *C. albicans*. To our knowledge, this is the first time that Nce103 has been implicated in cell wall remodeling, although Sch9 and Rca1 have previously been implicated in the regulation of cell wall genes (53, 55).

## DISCUSSION

β-1,3-Glucan exposure is a complex phenotype that is influenced by cell wall synthesis, the generation of maternal bud scars during cell division (17), β-1,3-glucan masking by the mannan outer layer of the cell wall (56), and the shaving of exposed β-1,3-glucan by secreted exoglucanases (17, 40). Mutations or drugs that perturb cell wall architecture (42, 43, 57, 58) and, in particular, mutations that compromise the outer mannan layer (56) can lead to β-1,3-glucan exposure at the cell surface. However, β-1,3-glucan masking does not correlate directly with changes in cell wall architecture, as masking has been observed on cell walls with significantly reduced outer mannan layers as well as those with significantly increased mannan layers (REFS). Furthermore, β-1,3-glucan exposure does not correlate with bulk levels of β-1,3-glucan (33, 34, 42).

Our exploration of β-1,3-glucan masking phenotypes across a well-defined set of *C. albicans* clinical isolates and the comparison of expression profiles in masking-competent and masking-defective isolates led to the identification of genes whose induction correlated with lactate-induced or hypoxia-induced masking. In *C. albicans,* β-1,3-glucan exposure is known to decrease during stationary phase (59), and therefore, our screen was performed on exponentially growing cells. The analysis of nine target genes highlighted by the screen identified two new loci that influence β-1,3-glucan masking in *C. albicans*.

The first locus, *PHO84*, encodes a high-affinity phosphate transporter and was necessary for lactate-induced masking. The basis for this apparent link between phosphate uptake and lactate-induced β-1,3-glucan masking is not yet clear. However, by analogy with iron limitation-induced β-1,3-glucan masking, which requires the iron transceptor, Ftr1 (34), the involvement of the Pho84 transporter might provide a clue that masking could potentially be induced by phosphate limitation. Alternatively, phosphate acquisition has been linked with metal bioavailability in *C. albicans* and with the expression of genes associated with iron assimilation (60). Therefore, *PHO84* inactivation could conceivably affect β-1,3-glucan exposure indirectly by perturbing iron acquisition and/or, potentially, by influencing cell morphology and growth (17, 61).

The second locus, *NCE103,* encodes carbonic anhydrase and was required for both lactate- and hypoxia-induced masking. Subsequent dissection revealed the involvement of the evolutionarily conserved Sch9-Rca1-Nce103 module (54) in β-1,3-glucan masking. On one hand, this made sense because cAMP-PKA signaling has been shown to play a key role in the regulation of β-1,3-glucan masking (33, 34). On the other hand, this was intriguing because the Nce103 has been reported to regulate responses to CO_2_ (50, 53, 54), and yet CO_2_ did not induce β-1,3-glucan masking in wild-type *C. albicans* cells (Fig. 6). Instead, Nce103 was required for β-1,3-glucan masking in response to both lactate and hypoxia. Interestingly, Sch9 has been shown to respond to additional inputs, including hypoxia (53, 55). Hence, the activity of the Sch9-Rca1-Nce103 module is likely to be modulated by multiple inputs.

**Fig 7:**
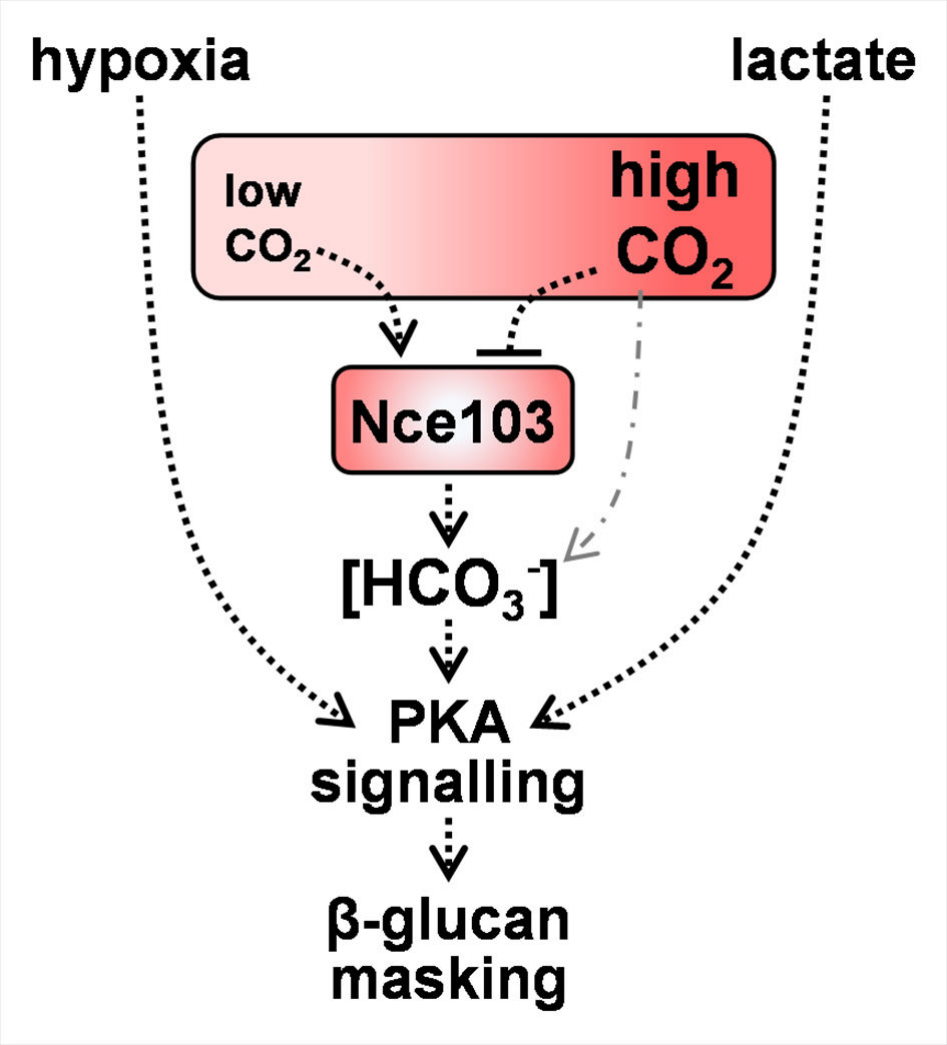
Model relating intracellular bicarbonate homeostasis and CO_2_ sensing via Nce103 to hypoxia- and lactate-induced β-1,3-glucan masking in *C. albicans*. Nce103 is a carbonic anhydrase that plays a critical role in the maintenance of intracellular bicarbonate homeostasis (HCO_3_^−^) and CO_2_ sensing. At low CO_2_ concentrations, Nce103 catalyzes the conversion of dissolved CO_2_ into bicarbonate (HCO_3_^−^) (50). At high CO_2_ concentrations, bicarbonate can be formed chemically from CO_2_ (dotted and dashed gray line), and consequently, Nce103 levels are downregulated by Sch9-mediated phosphorylation and inhibition of Rca1, a transcriptional activator of *NCE103* (53, 54). In addition to being used for C1 metabolism, bicarbonate interacts directly with adenylyl cyclase to stimulate the production of cAMP and thereby PKA signaling. Hence, normal growth at low CO_2_ levels is dependent upon Nce103, and the data indicate that the regulation of bicarbonate homeostasis by Nce103 appears to be essential for PKA-mediated regulation of lactate- or hypoxia-induced β-1,3-glucan masking in *C. albicans*.

If Nce103 is not required at high CO_2_ levels (50), why do not *rca1* and *nce103* cells display wild-type β-1,3-glucan masking phenotypes in the presence of high CO_2_ levels (Fig. 6A)? There is a straightforward explanation for this. The requirement for Nce103 at high CO_2_ levels was based on growth assays, not cell wall phenotypes (50). Therefore, Nce103 could conceivably be required for normal cell wall maintenance even at high CO_2_ levels. For example, aberrant bicarbonate homeostasis in *rca1* and *nce103* cells might influence adenylyl cyclase-cAMP-PKA signaling, thereby affecting β-1,3-glucan exposure under these conditions. In mammalian cells, bicarbonate binds directly to soluble adenylyl cyclase to induce a conformational change that promotes catalysis to form cAMP (62, 63). Homologs of these bicarbonate-responsive adenylyl cyclases are present in bacteria and fungi (50, 62), and it has been confirmed that purified adenylyl cyclase from *C. albicans* can be stimulated by bicarbonate *in vitro* (50). Klengel and colleagues have demonstrated that the reduced growth of *nce103* cells at low CO_2_ concentrations is not due to an insufficiency of substrates to support C1 metabolism (50). Instead, they suggest that this is due to a decrease in intracellular bicarbonate levels below the threshold required to activate adenylyl cyclase (50) and, consequently, those required for cAMP-PKA signaling. The fact that Nce103 is required for lactate- and hypoxia-induced β-1,3-glucan masking suggests that basal concentrations of intracellular bicarbonate may be required to permit activation of PKA-signaling-dependent processes in *C. albicans* such as β-1,3-glucan masking (Fig. 7) (33, 34). Equally, elevated bicarbonate levels might enhance β-1,3-glucan masking. Therefore, the Sch9-Rca1-Nce103 signaling module appears to drive homeostatic maintenance of the intracellular bicarbonate concentration by reducing carbonic anhydrase levels when ambient CO_2_ concentrations are high and by increasing them when ambient CO_2_ concentrations are low, thereby regulating PKA signaling (Fig. 7).

These findings are significant for the pathobiology of *C. albicans* and relevant to other fungal pathogens. The Sch9-Rca1-Nce103 CO_2_ sensing module promotes the growth and development of *C. albicans* and *Cryptococcus neoformans* (50, 64) and the virulence of *C. albicans* in niches containing low ambient CO_2_ concentrations (50). As an upstream regulator of PKA signaling, the Sch9-Rca1-Nce103 module influences yeast-hypha morphogenesis and resistance to cell wall stressors and antifungal drugs (50, 65) as well as β-1,3-glucan masking in *C. albicans* (Fig. 6). By promoting β-1,3-glucan masking, the Sch9-Rca1-Nce103 module contributes to an anticipatory protective response that, together with the inherent phenotypic variability in β-1,3-glucan exposure displayed by *C. albicans* (bet hedging), allows this fungus to evade immune recognition and the resultant antifungal immune responses (32–34, 38, 39, 66).

Is this specific PAMP masking strategy likely to be displayed by other pathogenic *Candida* species? The Sch9-Rca1-Nce103 signaling module is conserved in evolutionarily divergent yeasts (50, 53, 64, 67), and hypoxia-induced β-1,3-glucan masking has been observed in some other *Candida* pathogens: *Candida krusei* and *Candida tropicalis,* but not in *Candida glabrata, Candida parapsilosis,* or *Candida auris* (33). Therefore, even though anticipatory responses appear to be gained and lost relatively quickly in evolutionary terms (14, 68), it is conceivable that hypoxia-induced β-1,3-glucan masking might be regulated by the Sch9-Rca1-Nce103 module in a subset of *Candida* pathogens.

This PAMP masking strategy is one of several anticipatory responses that this human commensal has evolved. For example, *C. albicans* induces the expression of the pore-forming toxin candidalysin during hyphal development (69, 70), which is thought to anticipate nutrient limitation within the invasion pocket during tissue invasion (8, 13, 71). However, excessive candidalysin production induces tissue damage and triggers antifungal responses (69, 72). Likewise, elevated β-1,3-glucan exposure, particularly under acidic conditions (38, 39), might contribute to inflammation and tissue pathology during vaginitis (73) and modulate the levels of *C. albicans* colonization in the gastrointestinal tract (74, 75). Therefore, the complex interplay between these fungal anticipatory responses and the antifungal defenses of the host appears to permit the commensal lifestyle of *C. albicans* while constraining fungal outgrowth and infection (8, 69, 71, 72, 74, 76, 77).

## MATERIALS AND METHODS

### Strains and growth conditions

The laboratory strains of *C. albicans* are listed in Table S2 and the clinical isolates in Table S3. For analyses of β-1,3-glucan exposure, strains were grown overnight at 30°C at 200 rpm in minimal medium [GYNB: 2% glucose, 0.65% yeast nitrogen base without amino acids (78)] prepared with bottled water (Highland Spring, Blackford, UK). These cultures were then diluted in fresh, prewarmed GYNB to an OD_600_ of 0.2 and grown for 5 hours at 30°C at 200 rpm in normoxic GYNB (control), with 1% lactate (32), under hypoxia (33), under 5% CO_2_, or under a combination of hypoxia plus 5% CO_2_.

For analyses of stress and drug resistance, strains were plated on YPD [2% glucose, 2% mycological peptone, 1% yeast extract, and 2% agar (78)] containing the specified concentration of stressor, incubated at 30°C or at an alternative specified temperature, and imaged after 48 hours (79, 80).

### Strain construction

Homozygous null mutants were generated in *C. albicans* SC5314 using established CRISPR-Cas9 methodologies (81).

Briefly, the *CaCAS9* cassette was amplified from the plasmid pV1093 (82) using primers CaCas9/for and CaCas9/rev. The sgRNA cassette was constructed by PCR with the nested primers SNR52/N and sgRNA/N to fuse the DNA fragments comprising the *SNR52* promoter (amplified with primers SNR52/F and SNR52-sg-target-Rv) and the sgRNA scaffold (amplified with primers target-sg-scaf-Fw and sgRNA/R). Repair templates, which contained the *SAT1* marker and harbored 80-bp homology to the 5′ and 3′ ends of the target gene, were amplified from pV1093. PCR reactions were carried out using CloneAmp high-fidelity DNA polymerase in accordance with the manufacturer’s instructions (Clontech). Each unpurified PCR product (10 µL of CaCAS9 cassette, sgRNA cassette, and the relevant repair template) was transformed into *C. albicans* SC5314 using the lithium acetate transformation method (83). Transformants were selected on YPD containing 200 µg/mL nourseothricin (Jena Bioscience).

The disruption of both alleles of the target locus was confirmed by PCR (Fig. S2). The primers used for the construction and PCR diagnosis of these mutants are described in Table S7.

### Flow cytometry

Flow cytometry was used to quantify levels of β-1,3-glucan exposure on *C. albicans* cell populations (32–34). Cells were grown in GYNB at 30°C for 5 hours (above), fixed overnight with 50 mM thimerosal (Sigma-Aldrich, Dorset, UK), washed, and stained with Fc-Dectin-1 and anti-human IgG linked to Alexafluor 488 (Invitrogen). A BD Fortessa flow cytometer was used to record the fluorescence for 10,000 events per sample. A fixed gating strategy and axis scales were used throughout (Fig. S4). FlowJo v.10 software was used to quantify median fluorescence indices. Each cytometry plot is representative of at least three independent biological replicates. Fold changes in β-1,3-glucan exposure were calculated by dividing the MFI for the strain in question under the experimental condition (e.g., hypoxic GYNB) by the corresponding MFI for the control condition (e.g., normoxic GYNB). Mutants were always compared to their congenic “wild-type” parent in at least three independent experiments.

To quantify hypoxia- and lactate-induced β-1,3-glucan masking in the *C. albicans* clinical isolates, each isolate was grown in GYNB at 30°C overnight, subcultured into fresh medium (OD_600_ = 0.2), and grown for 5 hours, as described above: normoxic GYNB (control), hypoxic GYNB, or GYNB containing 1% lactate. These cells were then fixed with thimerosal, counted using a Neubauer hemocytometer, and 2.5 × 10^6^ cells added per well of a 96-well plate. The cells were then washed and stained with Fc-dectin-1 and the anti-human IgG-AF488 secondary antibody in 96-well format, and their fluorescence was quantified by flow cytometry using a MACSQuant analyzer (Miltenyi Biotec). MFIs and fold changes in β-1,3-glucan exposure were determined for three independent biological replicates, as described above.

### Microscopy

*C. albicans* cells were cultured in GYNB under the specified conditions (above), fixed with 50 mM thimerosal, and stained with Fc-dectin-1 and IgG-AF488 (β-1,3-glucan) and ConA-AF647 (mannan). High-resolution confocal microscopy was performed using a Zeiss LSM 880 microscope fitted with an alpha Plan-Apochromat 100×/1.46 Oil DIC objective. Images were taken in Airyscan fast mode to avoid sample bleaching. Post-image capture processing was performed using Zen Blue 2.3.

### RNA sequencing

*C. albicans* clinical isolates were selected for analysis based on their β-1,3-glucan masking phenotypes. The responsive isolates displayed masking in response to lactate and hypoxia (CEC3560, CEC3605, CEC3609, CEC4108, CEC4259), whereas the non-responsive isolates were defective in both lactate- and hypoxia-induced masking (CEC3534, CEC3544, CEC3621, CEC3636, CEC4035). Each strain was cultured in GYNB overnight, subcultured into fresh normoxic GYNB (control), hypoxic GYNB, or GYNB containing 1% lactate, as described above, grown for 1 hour at 30°C, then harvested for analysis, and frozen at −80°C. RNA was extracted from frozen cell pellets via Qiazol/chloroform extraction (Qiagen, UK) according to the manufacturer’s instructions, treated with TURBO DNAse (Ambion, Banchory, UK), and assessed using an Agilent 2100 Bioanalyzer.

RNA was prepared for sequencing using the Illumina TruSeq Stranded mRNA Kit following the manufacturer’s instructions. Sequencing was performed on three independent biological replicates for each condition using the High Output 1 × 75 Kit on the Illumina NextSeq500 platform. Raw fastq files were processed through FastQC (v. 0.11.8) and Trimgalore (v. 0.4.0), removing all reads with a phred score <20. Reads were aligned to the *C. albicans* SC5314 reference genome [www.candidagenome.org (84, 85)] using HISAT2 (v. 2.1.0), and alignments were processed with SAMtools (v.1.9). Aligned reads were quantified at gene regions using featureCounts (subread v. 5.0.1), utilizing the parameter to split multi-mapped reads as a fraction across all genes that they align to. Differential expression analysis was carried out using edgeR (version 3.16.5) on all genes with a count per million >1 in three or more samples, with a significance cutoff of adjusted *P* < 0.05. GO enrichment analysis was performed through the *Candida* Genome Database GO Term Finder.

### Statistical analyses

GraphPad Prism 9 was used for statistical analyses. Data were generated from at least three independent biological replicates and then expressed as means ± standard deviation. To test the statistical difference between two sets of data with a non-parametric distribution, we used one-way ANOVA (Tukey’s multiple comparison test). The following *P*-values were considered: *P < 0.05; **P < 0.01; ***P < 0.001; ****P < 0.0001.

## Data Availability

The authors declare that the data supporting the findings of this study are available within the paper (and the accompanying supplementary information files) or at EBI (www.ebi.ac.uk/arrayexpress: accession number E-MTAB-10986; data files at www.ebi.ac.uk/ena/browser/home, project PRJEB47705). The source data for Fig. 2 and 3 are available at EBI.

## SUPPLEMENTAL MATERIAL

Figure S1 mbio.01898-23-s0001.pdf β-1,3-Glucan exposure levels and growth of *C. albicans* clinical isolates.

Figure S2 mbio.01898-23-s0002.pdf Genotyping of *C. albicans* mutants.

Figure S3 mbio.01898-23-s0003.pdf Phenotyping of *C. albicans* mutants.

Figure S4 mbio.01898-23-s0004.pdf Gating strategy for flow cytometry experiments.

Tables S1 and S2 mbio.01898-23-s0005.pdf *C. albicans* clinical isolates and laboratory strains used in this study.

Tables S4 and S5 mbio.01898-23-s0006.pdf *C. albicans* genes induced in response to hypoxia and lactate.

Table S3 mbio.01898-23-s0007.xlsx Normalized RNAseq data.

Table S6 mbio.01898-23-s0008.pdf Stress and drug resistance of*C. albicans* mutants.

Table S7 mbio.01898-23-s0009.pdf β-1,3-Glucan exposure by *C. albicans* deletion mutants.

Table S8 mbio.01898-23-s0010.pdf Oligonucleotide primers used in this study.

ASM does not own the copyrights to Supplemental Material that may be linked to, or accessed through, an article. The authors have granted ASM a non-exclusive, world-wide license to publish the Supplemental Material files. Please contact the corresponding author directly for reuse.
