# Supplementary material for: A CO2 sensing module modulates β-1,3-glucan exposure in Candida albicans
Source: mBio. 2024 Jan 23;15(2):e01898-23. doi: 10.1128/mbio.01898-23 (PMC10865862; doi:10.1128/mbio.01898-23)
Supplement: Figure S1 — β-1,3-Glucan exposure levels and growth of C. albicans clinical isolates. [file mbio.01898-23-s0001.pdf]

Supplementary Figure S1

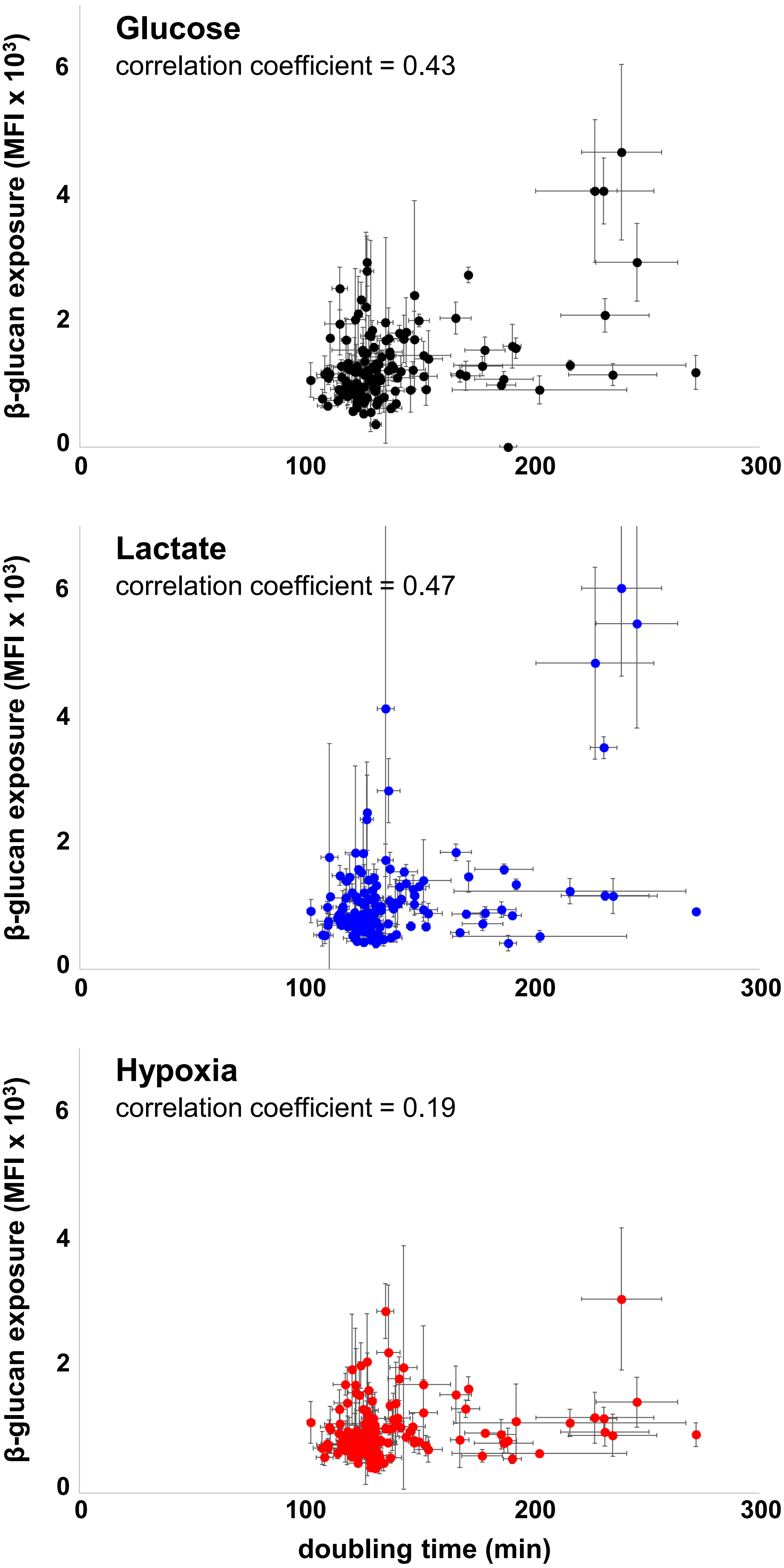

Supplementary Figure S1. β-1,3-glucan exposure levels and growth of *C. albicans* clinical isolates. Levels of β-1,3-glucan exposure displayed by clinical isolates were quantified by Fc-dectin-1 staining and flow cytometry during exponential growth either on GYNB (normoxic control), in the presence of lactate, or under hypoxia. Their growth on GYNB was measured in separate experiments (doubling time in min). Means and standard deviations are shown for three independent replicates.
