# Supplementary material for: A CO2 sensing module modulates β-1,3-glucan exposure in Candida albicans
Source: mBio. 2024 Jan 23;15(2):e01898-23. doi: 10.1128/mbio.01898-23 (PMC10865862; doi:10.1128/mbio.01898-23)
Supplement: Figure S2 — Genotyping of C. albicans mutants. [file mbio.01898-23-s0002.pdf]

Supplementary Figure S2

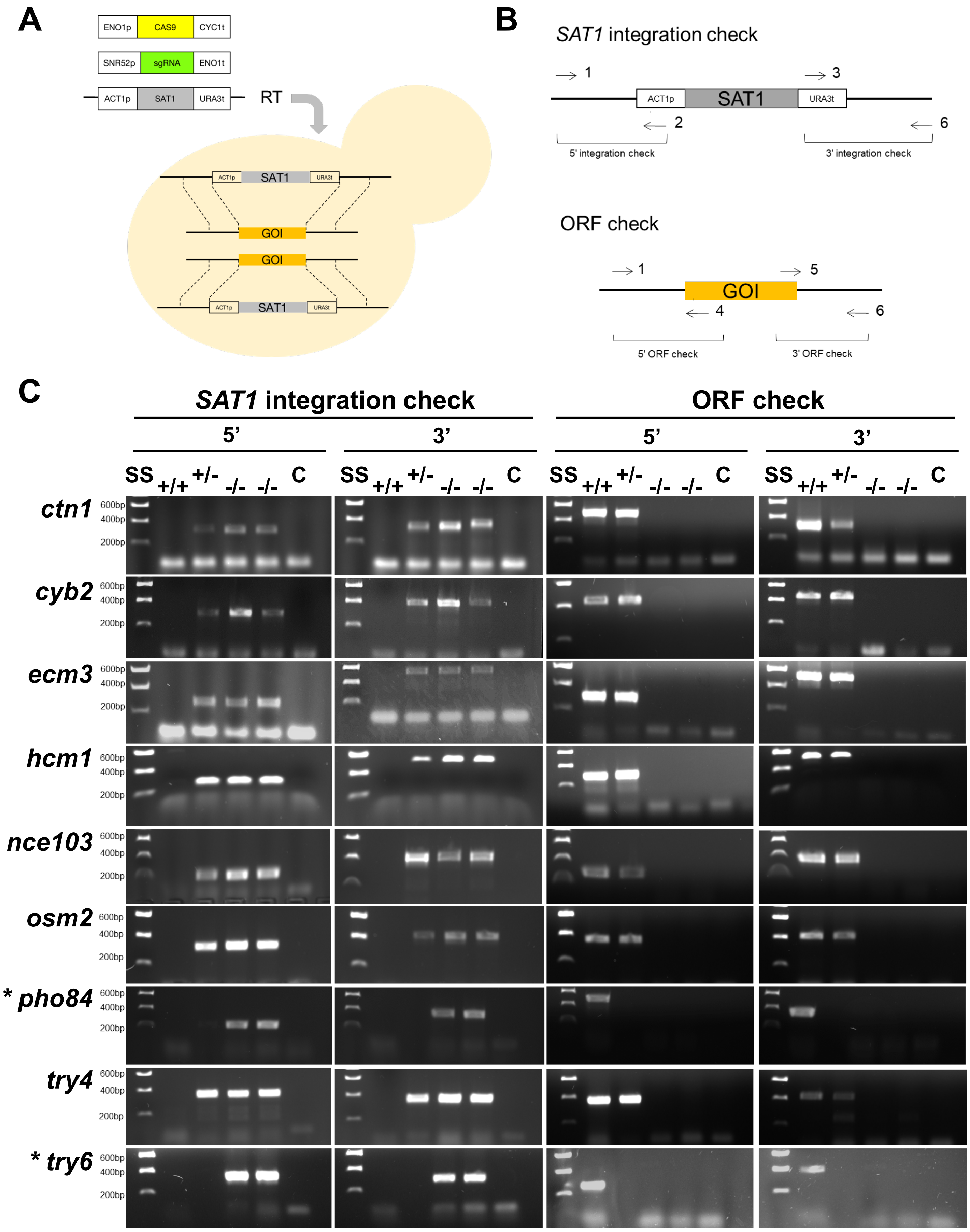

Supplementary Figure S2. Genotyping of *C. albicans* mutants. Homozygous null mutants were made in the *C. albicans* clinical isolate SC5314 using CRISPR-Cas technology. The primers used for mutant construction and PCR diagnosis are described in Supplementary Table 4. **(A)** The open reading frame of the target gene of interest (GOI) was deleted and replaced with a disruption cassette carrying the *SAT1* marker expressed from the *ACT1* promoter and *URA3* terminator. This was driven by *CAS9* expression from the *ENO1* promoter and *CYC1* terminator. **(B)** The genotypes of mutants were confirmed by diagnostic PCR using primers that checked integration of the *SAT* marker and loss of the target gene of interest (GOI) at both the 5' and 3' ends of the deletion/insertion. The approximate positions of the PCR primers, and their numbers are shown. **(C)** PCR confirmation of the mutants for the nine genes of interest (labelled to the left): SS, size standards, with their lengths shown by the left hand panels; +/+, SC5314 parental strain; +/-, heterozygous deletion mutant; -/-, homozygous deletion mutants A and B; C, control lacking template DNA. * No heterozygotes were identified for *PHO84* and *TRY6.*
