# Supplementary material for: A CO2 sensing module modulates β-1,3-glucan exposure in Candida albicans
Source: mBio. 2024 Jan 23;15(2):e01898-23. doi: 10.1128/mbio.01898-23 (PMC10865862; doi:10.1128/mbio.01898-23)
Supplement: Figure S3 — Phenotyping of C. albicans mutants. [file mbio.01898-23-s0003.pdf]

Supplementary Figure S3

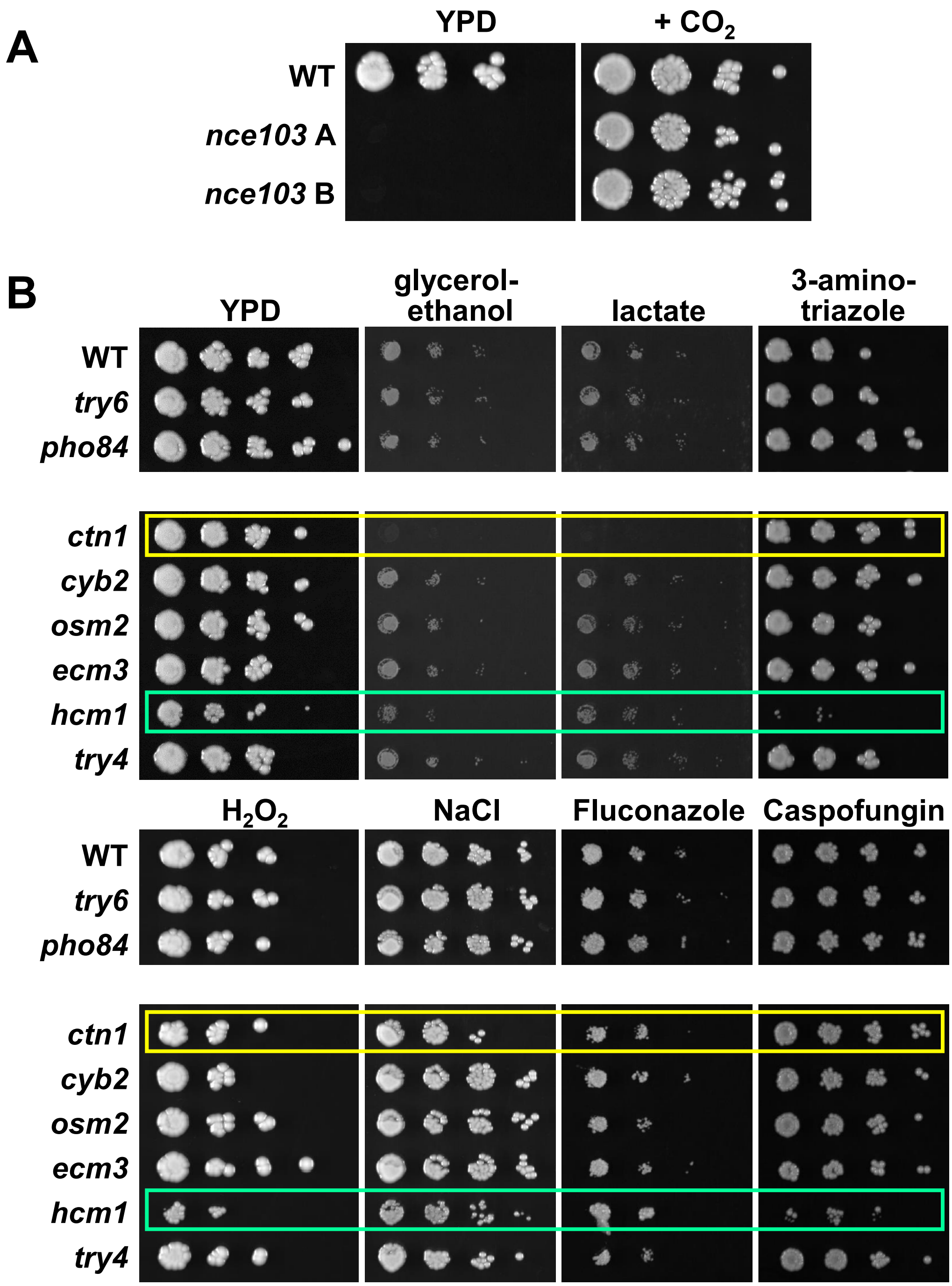

Supplementary Figure S3. Phenotyping of *C. albicans* mutants. The phenotypes of the homozygous null mutants were examined under a range of stress and nutrient conditions relative to the wild type parental strain SC5314 (WT) (Supplementary Table 5). A subset of representative experiments is presented here to highlight **(A)** the CO_2 auxotrophy of the *nce103* mutants, and **(B)** the poor growth of *ctn1* cells on nonfermentable carbon sources but not under other conditions tested (yellow boxes), and the slow growth of *hcm1* cells (green boxes). Series of 10-fold dilutions of overnight cultures were plated and the plates imaged after 48 h incubation at 30 °C: YPD control, 1% glycerol plus 1% ethanol; 2% lactate; 10 mM 3-aminotriazole (inducing amino acid starvation); 5 mM H_2O_2; 1 M NaCl; 0.65 µg/ml fluconazole; and 0.32 µg/ml caspofungin.
