# Supplementary material for: A CO2 sensing module modulates β-1,3-glucan exposure in Candida albicans
Source: mBio. 2024 Jan 23;15(2):e01898-23. doi: 10.1128/mbio.01898-23 (PMC10865862; doi:10.1128/mbio.01898-23)
Supplement: Figure S4 — Gating strategy for flow cytometry experiments. [file mbio.01898-23-s0004.pdf]

Supplementary Figure 4

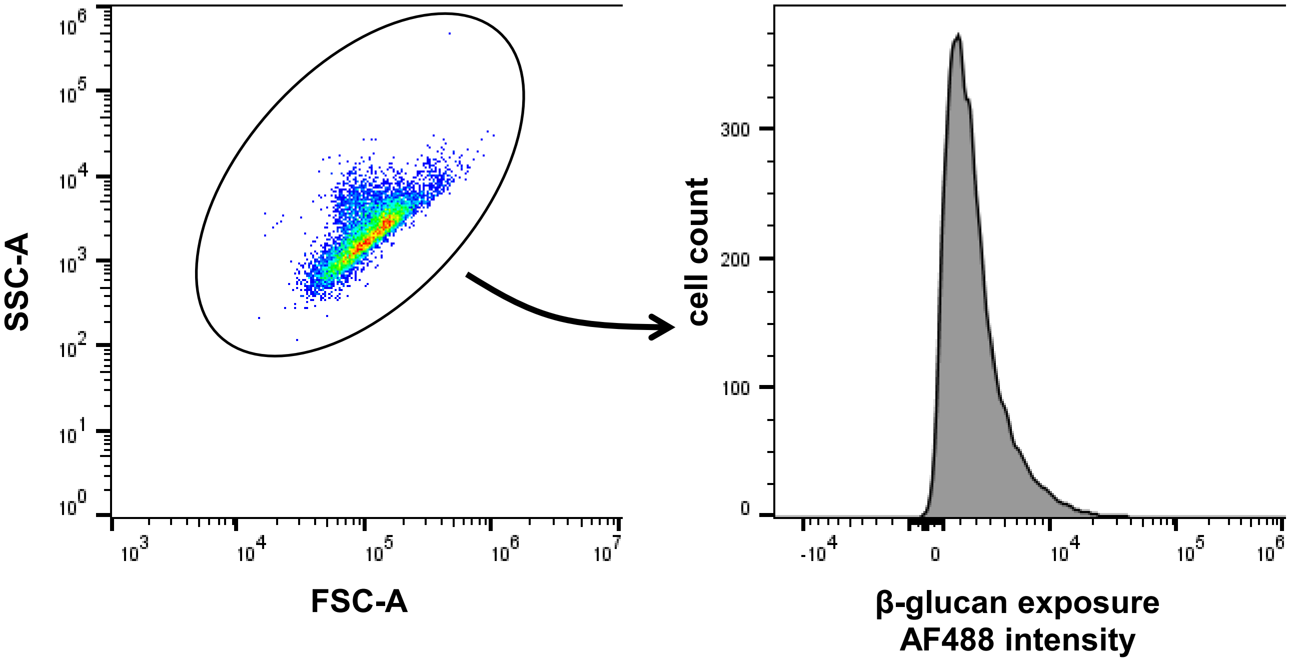

Supplementary Figure S4. Gating strategy for flow cytometry experiments. In all flow cytometry experiments, the *C. albicans* populations were gated on the basis of the scatter plot (FSC/SSC). The AF488 intensity of this gated population was then analysed. All the figures in the paper use the same axes as those shown in this figure: these axes were unchanged throughout.
