# Supplementary material for: A CO2 sensing module modulates β-1,3-glucan exposure in Candida albicans
Source: mBio. 2024 Jan 23;15(2):e01898-23. doi: 10.1128/mbio.01898-23 (PMC10865862; doi:10.1128/mbio.01898-23)
Supplement: Tables S1 and S2 — C. albicans clinical isolates and laboratory strains used in this study. [file mbio.01898-23-s0005.pdf]

Supplementary Table 1. *C. albicans* clinical isolates used in this study.

All isolates, with the exception of SC5314 (Supplementary Table 2), were from the Pasteur Institute.

Isolate Number	Cluster*	Infection	Isolate Number	Cluster*	Infection
SC5314	1	invasive			
CEC704	4	superficial	CEC3634	8	commensal
CEC708	4	superficial	CEC3636	1	commensal
CEC709	3	superficial	CEC3638	3	commensal
CEC711	4	superficial	CEC3658	1	superficial
CEC712	4	superficial	CEC3659	4	superficial
CEC716	12	superficial	CEC3660	1	superficial
CEC718	1	superficial	CEC3661	C	superficial
CEC1289	3	invasive	CEC3662	1	invasive
CEC1424	1	superficial	CEC3663	NC	invasive
CEC1426	11	superficial	CEC3668	9	superficial
CEC1427	4	invasive	CEC3669	2	superficial
CEC1492	4	unknown	CEC3672	1	invasive
CEC2018	9	superficial	CEC3673	1	invasive
CEC2019	12	superficial	CEC3674	4	superficial
CEC2020	1	superficial	CEC3675	4	invasive
CEC2021	2	invasive	CEC3676	4	superficial
CEC2022	4	invasive	CEC3678	1	superficial
CEC2023	8	commensal	CEC3679	1	superficial
CEC2871	18	invasive	CEC3680	12	invasive
CEC2872	18	invasive	CEC3681	3	invasive
CEC2876	18	invasive	CEC3682	1	invasive
CEC3494	11	superficial	CEC3683	4	invasive
CEC3529	1	commensal	CEC3685	12	invasive
CEC3530	4	commensal	CEC3686	12	invasive
CEC3531	2	commensal	CEC3704	11	superficial
CEC3532	4	commensal	CEC3711	10	superficial
CEC3533	9	commensal	CEC3712	NC	superficial
CEC3534	1	commensal	CEC3715	A	superficial
CEC3535	4	commensal	CEC3716	4	superficial
CEC3536	4	commensal	CEC3786	18	invasive
CEC3537	12	commensal	CEC4032	1	superficial
CEC3540	4	invasive	CEC4035	1	superficial
CEC3541	4	invasive	CEC4038	A	superficial
CEC3544	1	commensal	CEC4039	C	superficial
CEC3547	11	commensal	CEC4103	13	superficial
CEC3548	A	commensal	CEC4104	13	superficial
CEC3549	2	commensal	CEC4106	1	superficial
CEC3550	16	starling	CEC4107	1	superficial
CEC3551	4	superficial	CEC4108	1	superficial
CEC3553	2	commensal	CEC4256	1	superficial
CEC3554	3	superficial	CEC4259	1	invasive

CEC3555	NC	invasive	CEC4261	NC	superficial
CEC3556	4	commensal	CEC4479	1	environment
CEC3557	3	commensal	CEC4484	1	environment
CEC3558	2	commensal	CEC4487	2	environment
CEC3559	4	superficial	CEC4492	11	environment
CEC3560	1	superficial	CEC4497	4	environment
CEC3561	11	commensal	CEC4550	4	superficial
CEC3579	3	commensal	CEC4552	4	superficial
CEC3596	3	invasive	CEC4551	4	superficial
CEC3597	3	invasive	CEC4549	4	superficial
CEC3601	11	commensal	CEC4547	4	superficial
CEC3602	4	commensal	CEC4501	NC	superficial
CEC3603	1	commensal	CEC4502	NC	superficial
CEC3605	1	commensal	CEC4503	11	superficial
CEC3607	4	commensal	CEC4508	11	superficial
CEC3609	1	commensal	CEC4504	11	superficial
CEC3610	4	commensal	CEC4511	1	superficial
CEC3611	4	commensal	CEC4514	1	superficial
CEC3612	2	commensal	CEC4515	11	superficial
CEC3613	NC	commensal	CEC4519	11	superficial
CEC3614	2	commensal	CEC4520	11	superficial
CEC3615	2	commensal	CEC4521	11	superficial
CEC3616	10	commensal	CEC4522	11	superficial
CEC3617	1	commensal	CEC4523	11	superficial
CEC3618	11	commensal	CEC4524	11	superficial
CEC3619	NC	commensal	CEC4526	4	superficial
CEC3621	1	commensal	CEC4527	11	superficial
CEC3622	8	commensal	CEC4528	11	superficial
CEC3623	1	commensal	CEC4561	11	superficial
CEC3626	3	commensal	CEC4568	11	superficial
CEC3627	1	commensal	CEC4579	11	superficial

* NC, no cluster assigned

Supplementary Table 2. Laboratory strains of *C. albicans* used in this study.

Strain	pseudonym	Parent	Genotype	Source
SC5314			Clinical blood isolate	1
CAF2-1	CAF2-1	SC5314	*ura3/URA3*	2
CAI4	CAI4	CAF2-1	*ura3/ura3*	2
Ca372	CAI4+CIp10	CAI4	CAI4, *RPS1-CIp10 (URA3)*	3
SN152	SN152	CAF2-1	*arg4/arg4, leu2/leu2, his1/his1, URA3/ura3, IRO1/iro1*	4
Ca2469	*ctn1*-A	SC5314	*ctn1/ctn1*	this study
Ca2470	*ctn1*-B	SC5314	*ctn1/ctn1*	this study
Ca2467	*cyb2*-A	SC5314	*cyb2/cyb2*	this study
Ca2468	*cyb2*-B	SC5314	*cyb2/cyb2*	this study
Ca2465	*ecm3*-A	SC5314	*ecm3/ecm3*	this study
Ca2466	*ecm3*-B	SC5314	*ecm3/ecm3*	this study
Ca2475	*hcm1*-A	SC5314	*hcm1/hcm1*	this study
Ca2476	*hcm1*-B	SC5314	*hcm1/hcm1*	this study
Ca2481	*nce103*-A	SC5314	*nce103/nce103*	this study
Ca2482	*nce103*-B	SC5314	*nce103/nce103*	this study
Ca2472	*osm2*-A	SC5314	*osm2/osm2*	this study
Ca2473	*osm2*-B	SC5314	*osm2/osm2*	this study
Ca2486	*pho84*-A	SC5314	*pho84/pho84*	this study
Ca2487	*pho84*-B	SC5314	*pho84/pho84*	this study
Ca2478	*try4*-A	SC5314	*try4/try4*	this study
Ca2479	*try4*-B	SC5314	*try4/try4*	this study
Ca2484	*try6*-A	SC5314	*try6/try6*	this study
Ca2485	*try6*-B	SC5314	*try6/try6*	this study
CAS4	*sch9*	CAI4	CAI4, *sch9::hisG/sch9::hisG*	5
rca1ΔY	*rca1*	SN152	SN152, *sch9::HIS1/sch9::LEU2*	6

1. Gillum *et al.* (1984) Isolation of the *Candida albicans* gene for orotidine-5'-phosphate decarboxylase by complementation of *S. cerevisiae ura3* and *E. coli pyrF* mutations. *Molec. Gen. Genet*. 198, 179-182.
2. Fonzi & Irwin (1993) Isogenic strain construction and gene mapping in *Candida albicans*. *Genetics* 134, 717-728.
3. Murad *et al.* (2001) *NRG1* represses yeast-hypha morphogenesis and hypha-specific gene expression in *Candida albicans*. *EMBO. J.* 20, 4742-4752.
4. Noble & Johnson (2005) Strains and strategies for large-scale gene deletion studies of the diploid human fungal pathogen *Candida albicans*. *Eukaryotic Cell*. 4, 298-309.
5. Stichternoth *et al.* (2011) Sch9 kinase integrates hypoxia and CO_2 sensing to suppress hyphal morphogenesis in *Candida albicans*. *Eukaryotic Cell*. 10, 502–511.
6. Homann *et al.* (2009) A phenotypic profile of the *Candida albicans* regulatory network. *PLoS Genet* 5, e1000783.
