# Supplementary material for: A CO2 sensing module modulates β-1,3-glucan exposure in Candida albicans
Source: mBio. 2024 Jan 23;15(2):e01898-23. doi: 10.1128/mbio.01898-23 (PMC10865862; doi:10.1128/mbio.01898-23)
Supplement: Tables S4 and S5 — C. albicans genes induced in response to hypoxia and lactate. [file mbio.01898-23-s0006.pdf]

HYPOXIA: UP-REGULATED GENES

>2-fold UP
>4-fold UP
Targets

	RESPONDERS					NON-RESPONDERS					NON-R logFC
Gene	logFC	logCPM	LR	PValue	FDR	logFC	logCPM	LR	PValue	FDR	RESP log FC
PHO84	5.60	7.05	56.34	6.09E-14	1.06E-11	2.70	7.05	17.13	3.49E-05	3.49E-05	0.48
TRY6	4.67	3.15	45.74	1.35E-11	8.91E-10	3.34	3.15	26.59	2.52E-07	2.52E-07	0.72
SAP2	3.82	3.67	25.20	5.18E-07	7.17E-06	1.26	3.67	3.33	6.82E-02	6.82E-02	0.33
HWP1	3.70	-0.02	23.92	1.01E-06	1.29E-05	0.71	-0.02	1.02	3.12E-01	3.12E-01	0.19
JEN2	3.58	1.47	21.27	3.99E-06	4.28E-05	0.40	1.47	0.32	5.70E-01	5.70E-01	0.11
TRY4	3.55	-0.44	22.32	2.31E-06	2.71E-05	0.34	-0.44	0.23	6.30E-01	6.30E-01	0.10
DUR3	3.53	4.70	32.54	1.17E-08	2.87E-07	1.44	4.70	6.36	1.17E-02	1.17E-02	0.41
BTA1	3.30	-0.63	45.58	1.46E-11	9.50E-10	1.12	-0.63	5.57	1.83E-02	1.83E-02	0.34
PBR1	3.21	-0.16	20.88	4.89E-06	5.13E-05	2.21	-0.16	10.88	9.75E-04	9.75E-04	0.69
NCE103	3.11	4.01	64.02	1.23E-15	2.80E-13	0.86	4.01	5.60	1.80E-02	1.80E-02	0.28
SMM1	3.08	3.93	36.75	1.35E-09	4.73E-08	0.79	3.93	2.81	9.35E-02	9.35E-02	0.26
CTN1	2.98	3.14	44.89	2.08E-11	1.28E-09	0.54	3.14	1.71	1.91E-01	1.91E-01	0.18
DBP2	2.95	7.10	38.25	6.24E-10	2.41E-08	0.37	7.10	0.71	4.01E-01	4.01E-01	0.13
CDC6	2.84	1.65	50.29	1.33E-12	1.33E-10	1.52	1.65	15.63	7.69E-05	7.69E-05	0.53
PGA26	2.69	2.33	20.87	4.92E-06	5.15E-05	0.45	2.33	0.65	4.22E-01	4.22E-01	0.17
MAK16	2.64	5.56	17.03	3.68E-05	2.83E-04	0.75	5.56	1.55	2.13E-01	2.13E-01	0.29
CDC54	2.62	4.56	62.61	2.52E-15	5.30E-13	1.62	4.56	25.54	4.34E-07	4.34E-07	0.62
HCM1	2.54	2.88	51.53	7.06E-13	7.86E-11	1.52	2.88	19.73	8.91E-06	8.91E-06	0.60
NOP14	2.50	5.39	22.09	2.60E-06	2.96E-05	0.35	5.39	0.49	4.84E-01	4.84E-01	0.14
UGA6	2.47	0.20	24.03	9.51E-07	1.23E-05	1.68	0.20	11.75	6.10E-04	6.10E-04	0.68
DIM1	2.47	4.30	17.84	2.40E-05	1.99E-04	0.02	4.30	0.00	9.76E-01	9.76E-01	0.01
ENP2	2.37	5.00	16.35	5.26E-05	3.83E-04	0.43	5.00	0.58	4.46E-01	4.46E-01	0.18
XOG1	2.37	4.93	36.79	1.32E-09	4.68E-08	1.39	4.93	13.39	2.53E-04	2.53E-04	0.59
HIT1	2.32	1.99	46.00	1.18E-11	8.30E-10	0.19	1.99	0.32	5.74E-01	5.74E-01	0.08
PCL1	2.29	3.47	52.39	4.55E-13	5.59E-11	1.09	3.47	12.50	4.06E-04	4.06E-04	0.47
MCM3	2.27	5.79	51.11	8.71E-13	9.18E-11	0.91	5.79	8.86	2.91E-03	2.91E-03	0.40
REI1	2.21	5.33	14.93	1.12E-04	7.18E-04	0.04	5.33	0.00	9.45E-01	9.45E-01	0.02
DOT4	2.20	5.59	27.99	1.22E-07	2.10E-06	0.49	5.59	1.53	2.16E-01	2.16E-01	0.22
UGA4	2.18	2.83	18.80	1.45E-05	1.29E-04	1.50	2.83	9.21	2.40E-03	2.40E-03	0.68
MET3	2.18	7.99	17.94	2.27E-05	1.89E-04	0.21	7.99	0.18	6.75E-01	6.75E-01	0.09
CDC46	2.17	4.75	50.47	1.21E-12	1.23E-10	1.02	4.75	11.87	5.70E-04	5.70E-04	0.47
OFI1	2.15	-1.29	8.88	2.88E-03	1.06E-02	0.74	-1.29	1.04	3.07E-01	3.07E-01	0.34
CYS3	2.15	9.18	20.90	4.83E-06	5.08E-05	0.23	9.18	0.25	6.15E-01	6.15E-01	0.11
NOG1	2.13	6.71	13.23	2.76E-04	1.51E-03	0.20	6.71	0.12	7.28E-01	7.28E-01	0.09
KTI11	2.13	2.63	11.32	7.65E-04	3.51E-03	0.81	2.63	1.77	1.84E-01	1.84E-01	0.38
GIT1	2.11	0.08	23.42	1.30E-06	1.64E-05	0.33	0.08	0.60	4.38E-01	4.38E-01	0.16
NAN1	2.10	5.61	23.55	1.22E-06	1.54E-05	0.53	5.61	1.61	2.04E-01	2.04E-01	0.25
NIP7	2.09	4.90	12.25	4.65E-04	2.34E-03	0.07	4.90	0.02	8.99E-01	8.99E-01	0.03
OGG1	2.07	3.19	17.75	2.52E-05	2.07E-04	0.59	3.19	1.56	2.12E-01	2.12E-01	0.29
UTP13	2.07	5.71	15.61	7.80E-05	5.31E-04	0.30	5.71	0.37	5.46E-01	5.46E-01	0.15
RMS1	2.07	4.78	36.78	1.33E-09	4.68E-08	0.24	4.78	0.54	4.64E-01	4.64E-01	0.12
PZF1	2.06	3.20	40.77	1.71E-10	7.88E-09	0.74	3.20	5.60	1.80E-02	1.80E-02	0.36
HGT17	2.05	0.70	14.47	1.42E-04	8.68E-04	0.64	0.70	1.45	2.28E-01	2.28E-01	0.31
CAN1	2.05	6.49	31.65	1.84E-08	4.24E-07	0.48	6.49	1.88	1.71E-01	1.71E-01	0.23
NOC4	2.04	5.34	15.93	6.56E-05	4.58E-04	0.12	5.34	0.06	8.11E-01	8.11E-01	0.06
NOP6	2.03	4.83	18.25	1.94E-05	1.66E-04	0.08	4.83	0.03	8.67E-01	8.67E-01	0.04
KRR1	2.02	5.83	12.07	5.12E-04	2.54E-03	0.16	5.83	0.08	7.79E-01	7.79E-01	0.08
CCC1	2.02	6.05	21.76	3.09E-06	3.47E-05	0.92	6.05	4.79	2.86E-02	2.86E-02	0.46
NOG2	2.02	6.13	12.96	3.19E-04	1.70E-03	0.35	6.13	0.41	5.21E-01	5.21E-01	0.17
RRN11	2.00	3.40	29.08	6.95E-08	1.32E-06	0.55	3.40	2.30	1.29E-01	1.29E-01	0.27
ENP1	2.00	4.94	11.62	6.52E-04	3.10E-03	0.34	4.94	0.36	5.47E-01	5.47E-01	0.17
SPB4	1.99	4.41	15.74	7.27E-05	4.99E-04	0.20	4.41	0.17	6.77E-01	6.77E-01	0.10
NOP8	1.99	4.55	17.44	2.97E-05	2.38E-04	0.40	4.55	0.76	3.83E-01	3.83E-01	0.20
FRE10	1.98	9.13	38.37	5.86E-10	2.34E-08	0.58	9.13	3.56	5.91E-02	5.91E-02	0.29
ECM1	1.97	4.35	16.11	5.97E-05	4.23E-04	0.14	4.35	0.09	7.69E-01	7.69E-01	0.07
JIP5	1.97	5.22	16.48	4.93E-05	3.64E-04	0.21	5.22	0.21	6.50E-01	6.50E-01	0.11
TOP1	1.96	5.26	46.20	1.07E-11	7.69E-10	0.27	5.26	0.96	3.27E-01	3.27E-01	0.14
CFL5	1.96	5.58	14.54	1.37E-04	8.44E-04	0.03	5.58	0.00	9.50E-01	9.50E-01	0.02
MCM2	1.94	4.37	51.43	7.44E-13	8.12E-11	1.11	4.37	17.76	2.51E-05	2.51E-05	0.57
SPB1	1.94	5.80	12.39	4.32E-04	2.20E-03	0.14	5.80	0.07	7.93E-01	7.93E-01	0.07
SDA1	1.93	6.27	15.12	1.01E-04	6.64E-04	0.11	6.27	0.05	8.25E-01	8.25E-01	0.06
NSA1	1.93	5.60	10.51	1.18E-03	5.11E-03	0.25	5.60	0.20	6.59E-01	6.59E-01	0.13
MCM6	1.92	5.39	54.13	1.88E-13	2.46E-11	0.81	5.39	10.03	1.54E-03	1.54E-03	0.42
CIC1	1.91	5.91	9.81	1.74E-03	7.02E-03	0.48	5.91	0.67	4.13E-01	4.13E-01	0.25
ZCF4	1.91	0.09	11.94	5.49E-04	2.70E-03	0.65	0.09	1.41	2.35E-01	2.35E-01	0.34
SUL2	1.90	6.14	13.96	1.87E-04	1.09E-03	0.13	6.14	0.06	7.99E-01	7.99E-01	0.07

SET6	1.89	3.59	41.61	1.11E-10	5.60E-09	0.06	3.59	0.05	8.23E-01	8.23E-01	0.03
UTP4	1.89	5.73	11.19	8.24E-04	3.75E-03	0.17	5.73	0.09	7.60E-01	7.60E-01	0.09
CSI2	1.88	4.11	12.80	3.47E-04	1.82E-03	0.27	4.11	0.27	6.01E-01	6.01E-01	0.14
HCA4	1.88	5.85	11.30	7.75E-04	3.55E-03	0.08	5.85	0.02	8.88E-01	8.88E-01	0.04
DIP2	1.88	5.88	13.28	2.68E-04	1.47E-03	0.23	5.88	0.22	6.41E-01	6.41E-01	0.12
RRP9	1.88	5.27	12.14	4.95E-04	2.47E-03	0.49	5.27	0.89	3.45E-01	3.45E-01	0.26
PWP2	1.88	5.77	11.84	5.80E-04	2.83E-03	0.04	5.77	0.01	9.42E-01	9.42E-01	0.02
YTM1	1.87	5.84	10.11	1.47E-03	6.12E-03	0.19	5.84	0.11	7.42E-01	7.42E-01	0.10
SEN2	1.84	5.18	11.78	5.98E-04	2.88E-03	0.60	5.18	1.32	2.50E-01	2.50E-01	0.33
UGA11	1.84	3.56	91.02	1.42E-21	4.19E-18	0.82	3.56	18.96	1.34E-05	1.34E-05	0.45
BUD22	1.82	4.66	14.91	1.13E-04	7.22E-04	0.05	4.66	0.01	9.19E-01	9.19E-01	0.03
SAS10	1.82	5.52	9.83	1.72E-03	6.96E-03	0.24	5.52	0.18	6.75E-01	6.75E-01	0.13
RMP1	1.80	2.56	14.80	1.20E-04	7.56E-04	0.45	2.56	0.97	3.24E-01	3.24E-01	0.25
KRE30	1.79	7.24	16.09	6.04E-05	4.26E-04	0.64	7.24	2.18	1.40E-01	1.40E-01	0.36
HGT19	1.79	5.68	13.35	2.59E-04	1.43E-03	0.47	5.68	0.96	3.27E-01	3.27E-01	0.26
HGT10	1.78	4.82	7.72	5.46E-03	1.80E-02	0.99	4.82	2.49	1.14E-01	1.14E-01	0.56
TSR2	1.77	5.25	13.16	2.86E-04	1.55E-03	0.09	5.25	0.04	8.46E-01	8.46E-01	0.05
RCL1	1.77	5.02	16.38	5.18E-05	3.79E-04	0.19	5.02	0.19	6.61E-01	6.61E-01	0.11
UTP5	1.77	6.36	11.70	6.26E-04	2.99E-03	0.07	6.36	0.02	8.85E-01	8.85E-01	0.04
DBP3	1.77	6.18	9.27	2.33E-03	8.96E-03	0.15	6.18	0.07	7.89E-01	7.89E-01	0.09
RPP1	1.77	3.54	35.35	2.75E-09	8.49E-08	0.06	3.54	0.04	8.34E-01	8.34E-01	0.03
MCD1	1.76	4.06	30.57	3.22E-08	6.81E-07	1.27	4.06	16.30	5.41E-05	5.41E-05	0.72
MTG1	1.75	4.05	12.43	4.21E-04	2.16E-03	0.57	4.05	1.40	2.36E-01	2.36E-01	0.33
ERB1	1.75	5.48	17.22	3.33E-05	2.62E-04	0.18	5.48	0.19	6.65E-01	6.65E-01	0.10
HEM3	1.75	5.76	35.02	3.27E-09	9.75E-08	0.75	5.76	6.68	9.76E-03	9.76E-03	0.43
WOR3	1.75	3.68	21.54	3.46E-06	3.82E-05	0.03	3.68	0.01	9.39E-01	9.39E-01	0.02
UTP18	1.74	6.15	9.34	2.24E-03	8.64E-03	0.11	6.15	0.04	8.47E-01	8.47E-01	0.06
ACH1	1.74	7.15	73.06	1.26E-17	5.31E-15	0.76	7.15	14.54	1.38E-04	1.38E-04	0.44
HGT12	1.73	2.61	5.96	1.47E-02	3.97E-02	1.21	2.61	3.01	8.30E-02	8.30E-02	0.70
DPB2	1.72	3.50	22.06	2.64E-06	3.00E-05	0.66	3.50	3.39	6.57E-02	6.57E-02	0.38
RPC53	1.72	4.79	17.31	3.17E-05	2.51E-04	0.38	4.79	0.87	3.50E-01	3.50E-01	0.22
ELF1	1.71	7.24	12.02	5.28E-04	2.61E-03	0.25	7.24	0.27	6.03E-01	6.03E-01	0.15
BRG1	1.70	8.43	11.82	5.87E-04	2.84E-03	0.26	8.43	0.30	5.83E-01	5.83E-01	0.16
CWC22	1.68	3.78	21.29	3.94E-06	4.24E-05	0.62	3.78	2.99	8.40E-02	8.40E-02	0.37
OAC1	1.66	2.94	12.10	5.03E-04	2.51E-03	0.15	2.94	0.10	7.50E-01	7.50E-01	0.09
RDN5	1.66	3.04	7.53	6.08E-03	1.96E-02	1.88	3.04	9.53	2.02E-03	2.02E-03	1.13
SSF1	1.65	5.45	10.38	1.27E-03	5.43E-03	0.20	5.45	0.16	6.92E-01	6.92E-01	0.12
XUT1	1.65	3.69	33.49	7.15E-09	1.87E-07	1.09	3.69	14.92	1.12E-04	1.12E-04	0.66
DBP8	1.64	4.67	10.80	1.01E-03	4.48E-03	0.04	4.67	0.01	9.29E-01	9.29E-01	0.03
IMP4	1.64	5.61	11.46	7.11E-04	3.31E-03	0.28	5.61	0.36	5.49E-01	5.49E-01	0.17
RNY11	1.63	-0.08	25.91	3.58E-07	5.30E-06	0.98	-0.08	9.29	2.30E-03	2.30E-03	0.60
FYV5	1.62	3.30	12.51	4.05E-04	2.08E-03	0.04	3.30	0.01	9.37E-01	9.37E-01	0.02
RRP8	1.61	4.98	9.99	1.57E-03	6.44E-03	0.17	4.98	0.12	7.32E-01	7.32E-01	0.11
CHR1	1.61	5.12	17.03	3.68E-05	2.83E-04	0.25	5.12	0.43	5.11E-01	5.11E-01	0.16
TSR1	1.59	6.49	8.66	3.25E-03	1.17E-02	0.34	6.49	0.42	5.16E-01	5.16E-01	0.22
KTI12	1.58	4.70	8.38	3.79E-03	1.32E-02	0.04	4.70	0.00	9.47E-01	9.47E-01	0.02
PRN1	1.58	3.11	28.28	1.05E-07	1.84E-06	0.52	3.11	3.16	7.53E-02	7.53E-02	0.33
SMC3	1.58	3.33	25.52	4.38E-07	6.28E-06	0.89	3.33	8.43	3.69E-03	3.69E-03	0.57
ERG2	1.56	4.54	26.75	2.32E-07	3.68E-06	0.71	4.54	5.74	1.66E-02	1.66E-02	0.45
ALG11	1.56	3.61	21.81	3.01E-06	3.40E-05	0.78	3.61	5.72	1.68E-02	1.68E-02	0.50
VRG4	1.56	6.09	32.13	1.44E-08	3.45E-07	0.86	6.09	10.07	1.51E-03	1.51E-03	0.55
SEF2	1.56	5.19	15.81	6.99E-05	4.83E-04	1.01	5.19	6.89	8.67E-03	8.67E-03	0.65
CNS1	1.55	5.55	10.10	1.48E-03	6.13E-03	0.16	5.55	0.12	7.33E-01	7.33E-01	0.10
RLP24	1.54	5.70	6.67	9.81E-03	2.91E-02	0.05	5.70	0.01	9.28E-01	9.28E-01	0.03
DCC1	1.52	2.55	24.85	6.19E-07	8.37E-06	0.75	2.55	6.23	1.25E-02	1.25E-02	0.49
RPA135	1.52	7.00	8.00	4.68E-03	1.59E-02	0.04	7.00	0.01	9.33E-01	9.33E-01	0.03
LTV1	1.52	6.05	6.57	1.04E-02	3.02E-02	0.21	6.05	0.14	7.13E-01	7.13E-01	0.14
MLH1	1.52	2.00	39.73	2.91E-10	1.28E-08	0.88	2.00	13.73	2.11E-04	2.11E-04	0.58
PGA32	1.52	3.42	33.38	7.58E-09	1.97E-07	1.17	3.42	20.35	6.45E-06	6.45E-06	0.77
ESC4	1.52	2.88	20.38	6.34E-06	6.40E-05	0.72	2.88	4.74	2.95E-02	2.95E-02	0.47
NOP15	1.52	5.11	6.61	1.01E-02	2.98E-02	0.11	5.11	0.04	8.46E-01	8.46E-01	0.07
TRM1	1.51	4.94	9.21	2.41E-03	9.15E-03	0.53	4.94	1.17	2.80E-01	2.80E-01	0.35
HBR3	1.50	6.01	6.22	1.27E-02	3.54E-02	0.15	6.01	0.06	8.05E-01	8.05E-01	0.10
RRN3	1.50	6.05	7.64	5.72E-03	1.87E-02	0.11	6.05	0.05	8.31E-01	8.31E-01	0.08
ENA21	1.49	7.82	33.51	7.08E-09	1.86E-07	0.47	7.82	3.39	6.54E-02	6.54E-02	0.31
SRP40	1.49	6.61	5.75	1.65E-02	4.36E-02	0.45	6.61	0.54	4.64E-01	4.64E-01	0.30
CPA2	1.49	5.99	22.14	2.53E-06	2.93E-05	0.13	5.99	0.17	6.81E-01	6.81E-01	0.09
FDH1	1.49	4.75	7.72	5.46E-03	1.80E-02	0.65	4.75	1.50	2.21E-01	2.21E-01	0.43
RPA190	1.49	6.71	8.18	4.23E-03	1.46E-02	0.39	6.71	0.58	4.45E-01	4.45E-01	0.26
PUT1	1.49	3.55	12.75	3.56E-04	1.86E-03	0.81	3.55	3.90	4.82E-02	4.82E-02	0.55
SGD1	1.49	5.15	19.23	1.16E-05	1.06E-04	0.25	5.15	0.58	4.45E-01	4.45E-01	0.17
DRS1	1.47	6.26	7.73	5.43E-03	1.79E-02	0.20	6.26	0.15	6.97E-01	6.97E-01	0.14
OSM2	1.47	6.64	14.84	1.17E-04	7.42E-04	0.93	6.64	6.01	1.42E-02	1.42E-02	0.63

UTP21	1.47	5.79	7.80	5.23E-03	1.74E-02	0.03	5.79	0.00	9.56E-01	9.56E-01	0.02
CAT1	1.46	9.36	8.63	3.31E-03	1.19E-02	0.99	9.36	4.01	4.52E-02	4.52E-02	0.67
RLI1	1.46	7.70	7.23	7.19E-03	2.24E-02	0.67	7.70	1.56	2.12E-01	2.12E-01	0.46
CEM1	1.46	3.14	53.34	2.80E-13	3.59E-11	0.59	3.14	8.86	2.92E-03	2.92E-03	0.40
LAS1	1.45	4.76	26.82	2.24E-07	3.57E-06	0.29	4.76	1.15	2.84E-01	2.84E-01	0.20
CDC47	1.45	5.67	43.70	3.82E-11	2.15E-09	0.82	5.67	14.44	1.45E-04	1.45E-04	0.57
GUT1	1.45	5.23	18.04	2.16E-05	1.83E-04	0.29	5.23	0.74	3.90E-01	3.90E-01	0.20
PMS1	1.44	3.09	22.95	1.66E-06	2.04E-05	0.82	3.09	7.73	5.44E-03	5.44E-03	0.57
ALR1	1.43	6.93	28.15	1.12E-07	1.96E-06	0.47	6.93	3.10	7.83E-02	7.83E-02	0.33
NOC2	1.43	7.12	6.19	1.29E-02	3.57E-02	0.25	7.12	0.20	6.54E-01	6.54E-01	0.18
SMC1	1.42	4.83	26.44	2.72E-07	4.24E-06	0.60	4.83	4.78	2.88E-02	2.88E-02	0.42
GDA1	1.42	6.33	22.97	1.65E-06	2.03E-05	0.67	6.33	5.24	2.20E-02	2.20E-02	0.47
GIN1	1.41	4.25	19.55	9.82E-06	9.18E-05	0.56	4.25	3.17	7.50E-02	7.50E-02	0.40
RRP15	1.41	5.08	6.34	1.18E-02	3.36E-02	0.27	5.08	0.25	6.17E-01	6.17E-01	0.20
SWI6	1.41	4.69	36.62	1.43E-09	5.00E-08	0.80	4.69	12.14	4.93E-04	4.93E-04	0.57
RRP6	1.40	5.99	11.81	5.89E-04	2.85E-03	0.21	5.99	0.27	6.05E-01	6.05E-01	0.15
PRI2	1.40	3.14	13.21	2.79E-04	1.52E-03	0.61	3.14	2.54	1.11E-01	1.11E-01	0.43
RRP42	1.39	3.29	31.36	2.15E-08	4.79E-07	0.81	3.29	10.79	1.02E-03	1.02E-03	0.58
POP3	1.39	4.00	6.71	9.57E-03	2.85E-02	0.02	4.00	0.00	9.73E-01	9.73E-01	0.01
CHA1	1.36	4.07	16.58	4.66E-05	3.48E-04	0.99	4.07	8.84	2.94E-03	2.94E-03	0.72
MMS21	1.36	3.89	41.21	1.37E-10	6.45E-09	0.68	3.89	10.59	1.14E-03	1.14E-03	0.50
FMP45	1.34	6.07	17.32	3.16E-05	2.51E-04	0.60	6.07	3.51	6.10E-02	6.10E-02	0.44
MRT4	1.34	5.77	9.10	2.55E-03	9.61E-03	0.12	5.77	0.07	7.85E-01	7.85E-01	0.09
HYS2	1.34	5.02	16.00	6.34E-05	4.44E-04	0.36	5.02	1.22	2.70E-01	2.70E-01	0.27
NOP1	1.33	7.66	5.76	1.64E-02	4.36E-02	0.09	7.66	0.03	8.71E-01	8.71E-01	0.07
AMO2	1.32	6.26	6.17	1.30E-02	3.59E-02	0.34	6.26	0.42	5.16E-01	5.16E-01	0.26
RPC19	1.32	5.33	5.61	1.79E-02	4.63E-02	0.37	5.33	0.45	5.03E-01	5.03E-01	0.28
BUD21	1.32	5.46	11.92	5.55E-04	2.72E-03	0.01	5.46	0.00	9.80E-01	9.80E-01	0.01
RPC40	1.31	5.86	7.36	6.67E-03	2.09E-02	0.06	5.86	0.02	9.01E-01	9.01E-01	0.05
PWP1	1.31	6.62	5.66	1.73E-02	4.53E-02	0.05	6.62	0.01	9.32E-01	9.32E-01	0.04
RPF2	1.31	5.80	5.51	1.90E-02	4.84E-02	0.01	5.80	0.00	9.86E-01	9.86E-01	0.01
TPO4	1.30	5.06	49.50	1.99E-12	1.89E-10	0.64	5.06	12.08	5.11E-04	5.11E-04	0.49
PUS7	1.30	5.41	16.00	6.34E-05	4.44E-04	0.15	5.41	0.21	6.44E-01	6.44E-01	0.11
POL1	1.30	6.16	17.93	2.29E-05	1.90E-04	0.56	6.16	3.40	6.54E-02	6.54E-02	0.43
TRM2	1.29	5.47	5.76	1.64E-02	4.34E-02	0.36	5.47	0.45	5.02E-01	5.02E-01	0.28
SOD2	1.29	6.91	33.28	7.98E-09	2.05E-07	0.78	6.91	12.28	4.58E-04	4.58E-04	0.60
DCG1	1.28	2.77	15.15	9.91E-05	6.52E-04	0.62	2.77	3.57	5.87E-02	5.87E-02	0.48
YVH1	1.28	4.43	6.46	1.10E-02	3.18E-02	0.02	4.43	0.00	9.69E-01	9.69E-01	0.02
ANT1	1.27	2.81	30.64	3.11E-08	6.62E-07	0.38	2.81	2.81	9.39E-02	9.39E-02	0.30
ARG1	1.27	7.16	11.64	6.44E-04	3.07E-03	0.08	7.16	0.05	8.22E-01	8.22E-01	0.07
SKN1	1.27	2.05	28.58	8.98E-08	1.62E-06	0.83	2.05	12.33	4.47E-04	4.47E-04	0.65
MEA1	1.26	3.34	24.94	5.90E-07	8.02E-06	0.43	3.34	3.04	8.13E-02	8.13E-02	0.34
GUA1	1.26	7.79	5.93	1.49E-02	4.03E-02	0.25	7.79	0.24	6.26E-01	6.26E-01	0.20
MNT2	1.26	5.90	16.46	4.96E-05	3.65E-04	0.52	5.90	2.87	9.03E-02	9.03E-02	0.41
LAC1	1.26	5.27	15.89	6.70E-05	4.66E-04	0.54	5.27	2.96	8.52E-02	8.52E-02	0.43
SMC5	1.25	4.64	19.35	1.09E-05	1.01E-04	0.77	4.64	7.55	6.00E-03	6.00E-03	0.62
RIX7	1.25	5.37	15.19	9.74E-05	6.43E-04	0.28	5.37	0.80	3.71E-01	3.71E-01	0.23
FAS2	1.25	9.49	35.11	3.11E-09	9.32E-08	0.77	9.49	13.57	2.29E-04	2.29E-04	0.62
YOR1	1.25	5.85	11.26	7.90E-04	3.62E-03	0.59	5.85	2.57	1.09E-01	1.09E-01	0.47
THG1	1.24	3.33	41.34	1.28E-10	6.10E-09	0.39	3.33	4.06	4.39E-02	4.39E-02	0.31
MEP2	1.24	8.58	6.57	1.04E-02	3.03E-02	0.20	8.58	0.18	6.73E-01	6.73E-01	0.16
RAS2	1.23	4.08	10.21	1.40E-03	5.83E-03	0.19	4.08	0.25	6.15E-01	6.15E-01	0.15
BEM2	1.22	6.72	35.60	2.42E-09	7.51E-08	0.60	6.72	8.82	2.98E-03	2.98E-03	0.49
PEA2	1.22	5.31	21.57	3.42E-06	3.78E-05	0.39	5.31	2.21	1.37E-01	1.37E-01	0.32
DED1	1.22	7.45	9.36	2.22E-03	8.58E-03	0.26	7.45	0.45	5.02E-01	5.02E-01	0.22
MTG2	1.22	4.78	14.66	1.29E-04	8.03E-04	0.16	4.78	0.26	6.11E-01	6.11E-01	0.13
HEM1	1.21	7.16	27.19	1.85E-07	3.06E-06	0.33	7.16	2.09	1.49E-01	1.49E-01	0.27
DUR35	1.21	-0.64	6.66	9.87E-03	2.92E-02	0.92	-0.64	3.76	5.25E-02	5.25E-02	0.76
DBP7	1.21	5.59	10.35	1.29E-03	5.49E-03	0.06	5.59	0.02	8.81E-01	8.81E-01	0.05
UTP9	1.20	6.90	6.78	9.21E-03	2.76E-02	0.08	6.90	0.03	8.69E-01	8.69E-01	0.06
FAS1	1.20	9.67	35.14	3.07E-09	9.23E-08	0.72	9.67	12.82	3.43E-04	3.43E-04	0.60
MSH6	1.19	5.30	21.86	2.94E-06	3.32E-05	0.85	5.30	11.18	8.29E-04	8.29E-04	0.71
TIF3	1.19	7.76	9.22	2.40E-03	9.12E-03	0.45	7.76	1.32	2.50E-01	2.50E-01	0.37
FLU1	1.18	4.62	22.52	2.08E-06	2.48E-05	0.21	4.62	0.73	3.92E-01	3.92E-01	0.18
CTF8	1.18	1.68	10.02	1.55E-03	6.37E-03	0.47	1.68	1.65	1.99E-01	1.99E-01	0.40
AAH1	1.17	3.46	6.59	1.03E-02	3.01E-02	0.51	3.46	1.28	2.57E-01	2.57E-01	0.44
SMP3	1.17	3.66	14.74	1.23E-04	7.76E-04	0.48	3.66	2.53	1.12E-01	1.12E-01	0.41
ERG25	1.17	4.60	19.59	9.60E-06	9.05E-05	0.78	4.60	8.81	2.99E-03	2.99E-03	0.67
CIT1	1.16	8.14	12.53	4.01E-04	2.06E-03	0.22	8.14	0.46	5.00E-01	5.00E-01	0.19
GUK1	1.16	5.59	11.50	6.98E-04	3.27E-03	0.38	5.59	1.25	2.63E-01	2.63E-01	0.33
UTP22	1.16	6.25	8.53	3.50E-03	1.24E-02	0.42	6.25	1.16	2.82E-01	2.82E-01	0.36
MMS22	1.15	3.15	21.18	4.19E-06	4.47E-05	0.79	3.15	9.99	1.57E-03	1.57E-03	0.68
GIN4	1.15	5.42	36.49	1.54E-09	5.18E-08	0.76	5.42	16.10	6.00E-05	6.00E-05	0.66

QDR2	1.15	5.67	15.82	6.97E-05	4.82E-04	0.23	5.67	0.66	4.16E-01	4.16E-01	0.20
PRS1	1.15	6.05	8.47	3.62E-03	1.27E-02	0.24	6.05	0.38	5.40E-01	5.40E-01	0.21
DUT1	1.14	5.42	10.98	9.21E-04	4.14E-03	0.90	5.42	6.82	9.03E-03	9.03E-03	0.78
YBL053	1.13	4.15	19.93	8.03E-06	7.74E-05	0.57	4.15	5.10	2.39E-02	2.39E-02	0.50
ARG3	1.13	3.88	5.96	1.46E-02	3.97E-02	0.26	3.88	0.33	5.68E-01	5.68E-01	0.23
CRC1	1.13	5.53	29.99	4.35E-08	8.85E-07	0.54	5.53	7.02	8.06E-03	8.06E-03	0.48
ACS2	1.12	8.84	21.57	3.40E-06	3.78E-05	0.88	8.84	13.37	2.56E-04	2.56E-04	0.78
PSF3	1.12	2.72	14.58	1.34E-04	8.31E-04	0.59	2.72	4.08	4.33E-02	4.33E-02	0.52
DBF4	1.12	5.20	12.83	3.42E-04	1.81E-03	0.49	5.20	2.46	1.17E-01	1.17E-01	0.43
CSM3	1.12	3.96	15.01	1.07E-04	6.93E-04	0.55	3.96	3.65	5.60E-02	5.60E-02	0.49
GSC1	1.12	7.79	31.79	1.72E-08	4.04E-07	0.67	7.79	11.57	6.69E-04	6.69E-04	0.60
HXT5	1.11	5.04	13.44	2.47E-04	1.38E-03	0.31	5.04	1.04	3.07E-01	3.07E-01	0.28
HUT1	1.11	3.10	10.29	1.34E-03	5.63E-03	0.45	3.10	1.73	1.88E-01	1.88E-01	0.41
NMD5	1.11	6.08	11.99	5.35E-04	2.64E-03	0.32	6.08	0.99	3.19E-01	3.19E-01	0.28
ARG11	1.11	2.17	9.23	2.38E-03	9.08E-03	0.37	2.17	1.03	3.10E-01	3.10E-01	0.33
POP4	1.10	4.26	12.78	3.50E-04	1.84E-03	0.05	4.26	0.03	8.58E-01	8.58E-01	0.05
ASF1	1.10	4.03	18.47	1.72E-05	1.49E-04	0.60	4.03	5.56	1.84E-02	1.84E-02	0.54
URA7	1.09	7.26	6.56	1.04E-02	3.05E-02	0.06	7.26	0.02	8.87E-01	8.87E-01	0.05
RIO2	1.09	6.24	10.02	1.55E-03	6.36E-03	0.15	6.24	0.19	6.64E-01	6.64E-01	0.14
NMD3	1.09	6.64	6.25	1.24E-02	3.48E-02	0.05	6.64	0.02	9.01E-01	9.01E-01	0.05
SIT1	1.08	7.28	10.70	1.07E-03	4.71E-03	0.94	7.28	8.07	4.49E-03	4.49E-03	0.87
PEL1	1.08	5.48	36.00	1.97E-09	6.22E-08	0.25	5.48	1.94	1.64E-01	1.64E-01	0.23
ARG5,6	1.08	6.93	9.45	2.11E-03	8.23E-03	#N/A	#N/A	#N/A	#N/A	#N/A	#N/A
DDC1	1.07	3.23	30.13	4.04E-08	8.24E-07	0.64	3.23	10.81	1.01E-03	1.01E-03	0.59
PRP39	1.07	3.16	11.16	8.36E-04	3.80E-03	0.49	3.16	2.35	1.25E-01	1.25E-01	0.45
GCS1	1.07	6.28	18.84	1.42E-05	1.27E-04	0.15	6.28	0.38	5.37E-01	5.37E-01	0.14
NRM1	1.06	5.17	21.77	3.07E-06	3.45E-05	0.71	5.17	9.91	1.64E-03	1.64E-03	0.67
SPE2	1.06	6.41	9.77	1.77E-03	7.12E-03	0.38	6.41	1.26	2.62E-01	2.62E-01	0.36
ZCF19	1.06	2.66	17.99	2.22E-05	1.87E-04	0.30	2.66	1.49	2.22E-01	2.22E-01	0.28
MTS1	1.06	7.89	19.96	7.91E-06	7.69E-05	0.83	7.89	12.25	4.64E-04	4.64E-04	0.78
ZPR1	1.05	7.11	10.68	1.09E-03	4.76E-03	0.16	7.11	0.26	6.11E-01	6.11E-01	0.15
RFC4	1.05	5.56	9.97	1.60E-03	6.54E-03	0.49	5.56	2.18	1.40E-01	1.40E-01	0.46
HSX11	1.04	3.74	31.37	2.14E-08	4.79E-07	0.03	3.74	0.02	8.78E-01	8.78E-01	0.03
IRR1	1.04	5.06	15.17	9.85E-05	6.49E-04	0.69	5.06	6.75	9.37E-03	9.37E-03	0.66
OYE22	1.04	2.57	13.40	2.52E-04	1.40E-03	0.40	2.57	1.98	1.59E-01	1.59E-01	0.38
FAA21	1.04	4.52	52.73	3.82E-13	4.79E-11	0.61	4.52	18.24	1.95E-05	1.95E-05	0.59
CDR1	1.03	7.29	13.39	2.53E-04	1.40E-03	0.30	7.29	1.13	2.87E-01	2.87E-01	0.29
HMX1	1.03	8.11	6.18	1.29E-02	3.58E-02	0.94	8.11	5.13	2.35E-02	2.35E-02	0.91
MDR1	1.03	0.60	11.02	9.02E-04	4.07E-03	0.37	0.60	1.37	2.42E-01	2.42E-01	0.36
PGA46	1.03	1.38	18.67	1.55E-05	1.36E-04	0.56	1.38	5.57	1.83E-02	1.83E-02	0.54
PET127	1.03	4.99	5.71	1.69E-02	4.44E-02	0.28	4.99	0.43	5.11E-01	5.11E-01	0.27
NCS2	1.03	5.21	5.69	1.71E-02	4.48E-02	0.19	5.21	0.20	6.55E-01	6.55E-01	0.19
ERF1	1.03	7.55	7.96	4.78E-03	1.61E-02	0.41	7.55	1.26	2.62E-01	2.62E-01	0.39
GZF3	1.03	7.92	6.78	9.22E-03	2.76E-02	0.37	7.92	0.91	3.41E-01	3.41E-01	0.36
SLD5	1.02	2.48	25.01	5.69E-07	7.78E-06	0.67	2.48	10.97	9.27E-04	9.27E-04	0.66
GDH3	1.02	10.07	15.43	8.56E-05	5.74E-04	0.15	10.07	0.35	5.53E-01	5.53E-01	0.15
SMC6	1.02	5.41	12.95	3.20E-04	1.71E-03	0.32	5.41	1.29	2.57E-01	2.57E-01	0.31
PGA45	1.02	4.79	5.54	1.86E-02	4.77E-02	0.59	4.79	1.92	1.66E-01	1.66E-01	0.59
MNN4	1.01	5.77	19.24	1.15E-05	1.06E-04	0.45	5.77	3.88	4.89E-02	4.89E-02	0.45
ALK2	1.01	4.27	27.71	1.41E-07	2.42E-06	0.09	4.27	0.21	6.48E-01	6.48E-01	0.09
ACC1	1.01	8.73	21.67	3.23E-06	3.61E-05	0.69	8.73	10.10	1.48E-03	1.48E-03	0.68
RPR2	1.01	2.67	17.69	2.60E-05	2.12E-04	0.39	2.67	2.68	1.01E-01	1.01E-01	0.39
PRT1	1.00	7.96	5.61	1.79E-02	4.63E-02	0.47	7.96	1.23	2.67E-01	2.67E-01	0.46
NAG3	1.00	4.13	12.74	3.58E-04	1.87E-03	0.39	4.13	1.98	1.59E-01	1.59E-01	0.39
ENA2	1.00	6.02	9.06	2.61E-03	9.80E-03	1.04	6.02	9.84	1.71E-03	1.71E-03	1.04
YMX6	1.00	3.66	15.03	1.06E-04	6.85E-04	0.42	3.66	2.64	1.04E-01	1.04E-01	0.42
ADE8	0.99	6.21	10.22	1.39E-03	5.80E-03	0.23	6.21	0.57	4.51E-01	4.51E-01	0.23
INO2	0.98	3.79	24.56	7.20E-07	9.58E-06	0.21	3.79	1.10	2.94E-01	2.94E-01	0.21
UTP15	0.97	5.46	6.02	1.42E-02	3.86E-02	0.12	5.46	0.10	7.57E-01	7.57E-01	0.13
NTG1	0.97	4.54	11.51	6.93E-04	3.25E-03	0.37	4.54	1.66	1.97E-01	1.97E-01	0.38
HGT16	0.97	2.97	9.41	2.16E-03	8.38E-03	0.09	2.97	0.08	7.79E-01	7.79E-01	0.09
PEX3	0.97	4.65	38.65	5.07E-10	2.05E-08	0.42	4.65	7.48	6.22E-03	6.22E-03	0.44
TBF1	0.96	5.34	26.34	2.87E-07	4.42E-06	0.28	5.34	2.25	1.34E-01	1.34E-01	0.29
RFC5	0.96	4.32	11.02	9.00E-04	4.06E-03	0.45	4.32	2.45	1.18E-01	1.18E-01	0.47
DRE2	0.95	6.80	10.09	1.49E-03	6.17E-03	0.43	6.80	2.07	1.50E-01	1.50E-01	0.45
OPT8	0.94	5.02	14.45	1.44E-04	8.74E-04	0.18	5.02	0.52	4.72E-01	4.72E-01	0.19
CAT2	0.94	5.94	17.24	3.30E-05	2.60E-04	0.43	5.94	3.76	5.26E-02	5.26E-02	0.46
POL30	0.94	6.44	11.32	7.66E-04	3.52E-03	0.65	6.44	5.47	1.93E-02	1.93E-02	0.69
ROA1	0.93	5.55	22.62	1.97E-06	2.37E-05	0.21	5.55	1.20	2.73E-01	2.73E-01	0.23
RPB8	0.93	5.33	11.48	7.03E-04	3.28E-03	0.29	5.33	1.11	2.93E-01	2.93E-01	0.31
PUT2	0.92	4.91	12.09	5.06E-04	2.52E-03	0.11	4.91	0.17	6.81E-01	6.81E-01	0.12
CLA4	0.92	5.25	20.87	4.92E-06	5.15E-05	0.68	5.25	11.40	7.36E-04	7.36E-04	0.74
FAD2	0.91	7.65	8.39	3.77E-03	1.32E-02	0.53	7.65	2.85	9.16E-02	9.16E-02	0.58

PDE1	0.90	5.06	29.35	6.05E-08	1.18E-06	0.24	5.06	2.16	1.42E-01	1.42E-01	0.27
POL3	0.89	5.65	21.30	3.94E-06	4.24E-05	0.50	5.65	6.83	8.98E-03	8.98E-03	0.56
MRR1	0.89	4.93	26.67	2.41E-07	3.82E-06	0.58	4.93	11.36	7.52E-04	7.52E-04	0.65
GLR1	0.89	7.05	12.70	3.67E-04	1.91E-03	0.66	7.05	6.99	8.19E-03	8.19E-03	0.74
ISA1	0.88	7.56	13.98	1.85E-04	1.08E-03	0.15	7.56	0.43	5.13E-01	5.13E-01	0.17
SEC62	0.87	5.52	11.55	6.76E-04	3.18E-03	0.59	5.52	5.28	2.15E-02	2.15E-02	0.67
GPI7	0.87	4.26	12.17	4.85E-04	2.43E-03	0.42	4.26	2.82	9.31E-02	9.31E-02	0.48
CLG1	0.87	7.32	7.21	7.26E-03	2.25E-02	0.10	7.32	0.10	7.51E-01	7.51E-01	0.12
SAS2	0.87	3.96	5.83	1.57E-02	4.20E-02	0.45	3.96	1.56	2.12E-01	2.12E-01	0.51
SCH9	0.87	5.01	19.13	1.22E-05	1.11E-04	0.08	5.01	0.15	6.97E-01	6.97E-01	0.09
FOX3	0.86	4.72	16.52	4.81E-05	3.57E-04	0.55	4.72	6.75	9.37E-03	9.37E-03	0.64
FOX2	0.86	5.59	10.23	1.38E-03	5.80E-03	0.41	5.59	2.32	1.28E-01	1.28E-01	0.47
YMC2	0.86	5.27	7.37	6.63E-03	2.09E-02	0.28	5.27	0.80	3.72E-01	3.72E-01	0.33
PRN4	0.86	5.85	24.53	7.33E-07	9.71E-06	0.34	5.85	3.79	5.15E-02	5.15E-02	0.39
GDT1	0.86	5.41	36.33	1.67E-09	5.49E-08	0.19	5.41	1.77	1.83E-01	1.83E-01	0.22
UTP8	0.86	6.61	6.84	8.92E-03	2.69E-02	0.04	6.61	0.01	9.10E-01	9.10E-01	0.04
SEC14	0.86	7.37	14.53	1.38E-04	8.47E-04	0.03	7.37	0.02	8.78E-01	8.78E-01	0.04
PGA7	0.85	6.54	7.95	4.81E-03	1.62E-02	0.64	6.54	4.56	3.28E-02	3.28E-02	0.75
DNA2	0.84	4.40	16.01	6.32E-05	4.44E-04	0.36	4.40	2.94	8.65E-02	8.65E-02	0.43
POL5	0.83	4.61	10.41	1.26E-03	5.37E-03	0.02	4.61	0.00	9.44E-01	9.44E-01	0.02
HGT14	0.83	4.61	9.24	2.36E-03	9.03E-03	0.32	4.61	1.42	2.34E-01	2.34E-01	0.39
MEX67	0.83	6.59	24.71	6.68E-07	8.95E-06	0.30	6.59	3.19	7.40E-02	7.40E-02	0.36
ALA1	0.83	7.42	7.01	8.09E-03	2.48E-02	0.26	7.42	0.67	4.12E-01	4.12E-01	0.31
PTK2	0.83	6.19	49.67	1.82E-12	1.76E-10	0.32	6.19	7.37	6.63E-03	6.63E-03	0.38
ISU1	0.82	7.11	10.42	1.24E-03	5.33E-03	0.44	7.11	3.00	8.31E-02	8.31E-02	0.53
YOX1	0.82	5.45	9.49	2.07E-03	8.09E-03	0.58	5.45	4.79	2.86E-02	2.86E-02	0.71
PDS5	0.82	5.31	17.32	3.17E-05	2.51E-04	0.46	5.31	5.45	1.95E-02	1.95E-02	0.56
HMG1	0.81	6.78	10.86	9.82E-04	4.35E-03	0.58	6.78	5.53	1.86E-02	1.86E-02	0.71
PRP45	0.80	3.90	20.32	6.56E-06	6.55E-05	0.08	3.90	0.22	6.42E-01	6.42E-01	0.10
RGS2	0.80	4.37	10.74	1.05E-03	4.63E-03	0.06	4.37	0.07	7.93E-01	7.93E-01	0.08
CIS2	0.80	4.52	8.52	3.50E-03	1.24E-02	0.30	4.52	1.22	2.69E-01	2.69E-01	0.38
GLN4	0.80	6.59	13.35	2.58E-04	1.43E-03	0.39	6.59	3.25	7.14E-02	7.14E-02	0.49
DIP5	0.80	7.18	11.45	7.15E-04	3.32E-03	0.08	7.18	0.12	7.33E-01	7.33E-01	0.10
SKN2	0.79	5.25	7.44	6.39E-03	2.03E-02	0.50	5.25	2.98	8.45E-02	8.45E-02	0.63
EMP70	0.78	6.65	20.54	5.83E-06	5.99E-05	0.22	6.65	1.61	2.04E-01	2.04E-01	0.28
SNL1	0.78	5.40	12.99	3.14E-04	1.68E-03	0.76	5.40	12.25	4.65E-04	4.65E-04	0.97
TCC1	0.78	7.78	9.24	2.37E-03	9.05E-03	0.10	7.78	0.16	6.90E-01	6.90E-01	0.13
NUP84	0.77	5.53	17.70	2.58E-05	2.12E-04	0.25	5.53	1.86	1.72E-01	1.72E-01	0.32
SNM1	0.77	4.51	21.58	3.39E-06	3.77E-05	0.18	4.51	1.16	2.81E-01	2.81E-01	0.23
TEN1	0.76	1.79	6.56	1.05E-02	3.05E-02	0.06	1.79	0.04	8.39E-01	8.39E-01	0.08
HBR1	0.76	6.01	19.34	1.09E-05	1.01E-04	0.01	6.01	0.01	9.38E-01	9.38E-01	0.02
CCN1	0.76	4.02	10.15	1.44E-03	6.02E-03	0.63	4.02	6.98	8.25E-03	8.25E-03	0.83
IDI1	0.76	6.29	5.83	1.57E-02	4.20E-02	0.37	6.29	1.39	2.38E-01	2.38E-01	0.49
FAA2-3	0.76	3.25	25.26	5.01E-07	7.00E-06	0.68	3.25	19.98	7.83E-06	7.83E-06	0.89
NUP188	0.76	6.01	22.94	1.67E-06	2.04E-05	0.63	6.01	16.04	6.19E-05	6.19E-05	0.83
EDC3	0.76	5.26	12.68	3.69E-04	1.92E-03	0.18	5.26	0.74	3.89E-01	3.89E-01	0.24
PHO86	0.76	5.37	8.81	3.00E-03	1.10E-02	0.21	5.37	0.70	4.01E-01	4.01E-01	0.28
LYS21	0.75	6.53	11.80	5.91E-04	2.86E-03	0.31	6.53	1.96	1.62E-01	1.62E-01	0.41
CTF18	0.75	4.47	6.48	1.09E-02	3.15E-02	0.35	4.47	1.41	2.35E-01	2.35E-01	0.46
REX3	0.75	3.44	6.18	1.29E-02	3.58E-02	0.14	3.44	0.21	6.44E-01	6.44E-01	0.18
SOF1	0.75	6.26	5.55	1.85E-02	4.76E-02	0.11	6.26	0.12	7.24E-01	7.24E-01	0.15
WSC1	0.74	4.83	12.37	4.36E-04	2.22E-03	0.19	4.83	0.83	3.62E-01	3.62E-01	0.26
MAS2	0.74	5.81	6.35	1.18E-02	3.36E-02	0.10	5.81	0.11	7.38E-01	7.38E-01	0.13
CYS4	0.74	7.17	8.14	4.33E-03	1.49E-02	0.22	7.17	0.71	4.00E-01	4.00E-01	0.29
ADR1	0.74	4.48	7.83	5.15E-03	1.71E-02	0.12	4.48	0.20	6.52E-01	6.52E-01	0.16
RNR1	0.73	7.70	9.24	2.37E-03	9.05E-03	0.46	7.70	3.70	5.45E-02	5.45E-02	0.63
SEF1	0.73	6.55	10.41	1.25E-03	5.35E-03	0.35	6.55	2.37	1.24E-01	1.24E-01	0.48
ARO7	0.73	5.00	28.57	9.04E-08	1.63E-06	0.28	5.00	4.26	3.91E-02	3.91E-02	0.38
HFL1	0.73	4.12	20.29	6.66E-06	6.63E-05	0.50	4.12	9.60	1.95E-03	1.95E-03	0.69
SIR2	0.72	3.14	12.57	3.91E-04	2.02E-03	0.09	3.14	0.21	6.51E-01	6.51E-01	0.13
SAP10	0.72	5.80	16.57	4.69E-05	3.50E-04	0.49	5.80	7.63	5.73E-03	5.73E-03	0.68
MCD4	0.72	3.66	8.28	4.01E-03	1.39E-02	0.47	3.66	3.53	6.01E-02	6.01E-02	0.65
SMI1B	0.72	4.54	6.81	9.07E-03	2.72E-02	0.08	4.54	0.09	7.65E-01	7.65E-01	0.11
ANP1	0.72	5.20	8.94	2.79E-03	1.04E-02	0.50	5.20	4.40	3.58E-02	3.58E-02	0.70
NDT80	0.72	7.52	6.49	1.09E-02	3.14E-02	0.06	7.52	0.05	8.32E-01	8.32E-01	0.08
EXO1	0.71	4.90	11.61	6.55E-04	3.11E-03	0.49	4.90	5.53	1.87E-02	1.87E-02	0.69
RBR2	0.71	2.44	7.69	5.54E-03	1.82E-02	0.58	2.44	5.12	2.37E-02	2.37E-02	0.82
CDC13	0.71	3.89	15.71	7.38E-05	5.05E-04	0.40	3.89	5.10	2.40E-02	2.40E-02	0.57
PHO113	0.71	4.74	6.91	8.56E-03	2.60E-02	0.28	4.74	1.12	2.90E-01	2.90E-01	0.40
MLT1	0.71	7.44	7.65	5.68E-03	1.86E-02	0.37	7.44	2.08	1.49E-01	1.49E-01	0.52
SUI1	0.70	7.53	25.03	5.64E-07	7.74E-06	0.09	7.53	0.41	5.20E-01	5.20E-01	0.13
CHS3	0.70	7.44	6.36	1.17E-02	3.33E-02	0.23	7.44	0.72	3.96E-01	3.96E-01	0.34
POT1	0.69	5.54	5.78	1.62E-02	4.31E-02	0.55	5.54	3.71	5.41E-02	5.41E-02	0.80

MSH2	0.68	5.84	14.31	1.55E-04	9.29E-04	0.50	5.84	7.52	6.11E-03	6.11E-03	0.72
ZCF17	0.68	5.26	7.43	6.43E-03	2.04E-02	0.03	5.26	0.02	8.91E-01	8.91E-01	0.05
ABC1	0.68	7.24	6.22	1.26E-02	3.54E-02	0.14	7.24	0.27	6.02E-01	6.02E-01	0.21
GLO2	0.68	6.38	10.00	1.57E-03	6.42E-03	0.51	6.38	5.57	1.83E-02	1.83E-02	0.74
ISY1	0.68	3.84	6.98	8.22E-03	2.51E-02	0.19	3.84	0.54	4.61E-01	4.61E-01	0.28
PTC2	0.68	7.30	10.35	1.30E-03	5.49E-03	0.30	7.30	2.00	1.58E-01	1.58E-01	0.44
CAR1	0.67	5.71	5.68	1.71E-02	4.49E-02	0.23	5.71	0.67	4.13E-01	4.13E-01	0.34
FEN1	0.67	5.53	5.54	1.86E-02	4.77E-02	0.02	5.53	0.00	9.49E-01	9.49E-01	0.03
SPF1	0.67	7.08	9.83	1.71E-03	6.94E-03	0.47	7.08	4.93	2.64E-02	2.64E-02	0.71
SMD2	0.67	3.59	10.30	1.33E-03	5.61E-03	0.31	3.59	2.19	1.38E-01	1.38E-01	0.46
LEU5	0.67	5.56	8.10	4.43E-03	1.52E-02	0.10	5.56	0.17	6.82E-01	6.82E-01	0.14
POX1-3	0.66	6.34	8.88	2.88E-03	1.06E-02	0.26	6.34	1.38	2.40E-01	2.40E-01	0.39
TRX2	0.66	3.18	11.54	6.80E-04	3.20E-03	0.01	3.18	0.00	9.60E-01	9.60E-01	0.01
RTA3	0.65	5.73	12.58	3.90E-04	2.01E-03	0.25	5.73	1.88	1.71E-01	1.71E-01	0.39
EMP24	0.65	6.32	10.90	9.61E-04	4.28E-03	0.66	6.32	11.17	8.32E-04	8.32E-04	1.01
IFA21	0.65	5.33	20.68	5.42E-06	5.61E-05	0.59	5.33	17.27	3.25E-05	3.25E-05	0.91
UTR2	0.64	7.16	7.59	5.88E-03	1.91E-02	0.31	7.16	1.80	1.80E-01	1.80E-01	0.49
NMT1	0.64	5.82	13.60	2.26E-04	1.29E-03	0.35	5.82	4.05	4.43E-02	4.43E-02	0.54
PDC2	0.64	5.18	7.51	6.13E-03	1.97E-02	0.22	5.18	0.86	3.52E-01	3.52E-01	0.34
RAT1	0.64	5.97	10.49	1.20E-03	5.16E-03	0.16	5.97	0.63	4.28E-01	4.28E-01	0.24
GCN3	0.64	5.96	22.75	1.85E-06	2.23E-05	0.27	5.96	4.16	4.15E-02	4.15E-02	0.43
FAL1	0.64	5.72	6.98	8.26E-03	2.52E-02	0.20	5.72	0.67	4.14E-01	4.14E-01	0.31
MED1	0.63	5.20	20.70	5.36E-06	5.56E-05	0.46	5.20	10.93	9.44E-04	9.44E-04	0.73
GIR2	0.62	5.39	7.07	7.83E-03	2.41E-02	0.15	5.39	0.39	5.34E-01	5.34E-01	0.23
CAS4	0.62	6.49	13.40	2.51E-04	1.40E-03	0.44	6.49	6.64	9.97E-03	9.97E-03	0.70
RFC3	0.62	5.03	6.99	8.18E-03	2.50E-02	0.39	5.03	2.82	9.30E-02	9.30E-02	0.63
GUT2	0.62	8.36	5.82	1.58E-02	4.22E-02	0.26	8.36	1.03	3.11E-01	3.11E-01	0.42
MNN9	0.62	6.33	15.30	9.19E-05	6.12E-04	0.37	6.33	5.42	1.99E-02	1.99E-02	0.59
IML1	0.61	5.77	17.91	2.31E-05	1.92E-04	0.48	5.77	11.00	9.11E-04	9.11E-04	0.78
CHO1	0.60	5.94	7.08	7.81E-03	2.41E-02	0.00	5.94	0.00	9.97E-01	9.97E-01	0.00
CZF1	0.60	5.50	5.52	1.88E-02	4.81E-02	0.27	5.50	1.09	2.95E-01	2.95E-01	0.44
RCN1	0.60	2.64	7.01	8.09E-03	2.48E-02	0.42	2.64	3.37	6.65E-02	6.65E-02	0.70
SWI4	0.59	5.80	12.08	5.09E-04	2.53E-03	0.35	5.80	4.24	3.94E-02	3.94E-02	0.59
TES1	0.59	3.30	16.29	5.44E-05	3.94E-04	0.28	3.30	3.66	5.57E-02	5.57E-02	0.47
YEA4	0.59	3.16	18.58	1.63E-05	1.42E-04	0.34	3.16	6.09	1.36E-02	1.36E-02	0.57
HFL2	0.59	5.35	14.94	1.11E-04	7.15E-04	0.06	5.35	0.15	7.02E-01	7.02E-01	0.10
FGR13	0.59	3.37	12.12	4.99E-04	2.49E-03	0.34	3.37	3.99	4.57E-02	4.57E-02	0.58
GNP3	0.59	4.56	18.20	1.99E-05	1.70E-04	0.21	4.56	2.42	1.20E-01	1.20E-01	0.36
ASH2	0.58	5.41	19.07	1.26E-05	1.14E-04	0.32	5.41	5.69	1.70E-02	1.70E-02	0.55
UBA2	0.58	5.93	18.73	1.51E-05	1.33E-04	0.27	5.93	4.03	4.48E-02	4.48E-02	0.46
STE18	0.58	2.54	7.83	5.13E-03	1.71E-02	0.08	2.54	0.13	7.17E-01	7.17E-01	0.13
SOK1	0.58	7.41	10.85	9.86E-04	4.36E-03	0.13	7.41	0.53	4.66E-01	4.66E-01	0.22
POL2	0.57	5.54	6.21	1.27E-02	3.55E-02	0.41	5.54	3.22	7.28E-02	7.28E-02	0.72
MPH1	0.57	4.25	8.53	3.48E-03	1.24E-02	0.39	4.25	3.92	4.78E-02	4.78E-02	0.68
ROD1	0.57	6.93	7.06	7.88E-03	2.42E-02	0.01	6.93	0.00	9.69E-01	9.69E-01	0.01
ZCF35	0.57	4.33	13.94	1.89E-04	1.10E-03	0.11	4.33	0.53	4.66E-01	4.66E-01	0.20
NAT2	0.56	5.57	9.23	2.38E-03	9.07E-03	0.44	5.57	5.66	1.73E-02	1.73E-02	0.78
POL32	0.56	5.35	5.63	1.77E-02	4.60E-02	0.30	5.35	1.62	2.03E-01	2.03E-01	0.54
PBS2	0.56	6.99	8.51	3.53E-03	1.25E-02	0.26	6.99	1.91	1.67E-01	1.67E-01	0.47
CDC68	0.56	6.99	21.37	3.78E-06	4.13E-05	0.48	6.99	15.99	6.38E-05	6.38E-05	0.86
STE13	0.55	4.36	9.65	1.89E-03	7.53E-03	0.74	4.36	17.21	3.35E-05	3.35E-05	1.34
CDS1	0.55	6.66	33.96	5.63E-09	1.53E-07	0.10	6.66	1.23	2.68E-01	2.68E-01	0.19
SIM1	0.55	9.57	5.87	1.54E-02	4.13E-02	0.14	9.57	0.36	5.47E-01	5.47E-01	0.25
ECM14	0.54	6.54	16.15	5.85E-05	4.17E-04	0.20	6.54	2.21	1.37E-01	1.37E-01	0.37
HOL1	0.54	3.30	6.72	9.55E-03	2.85E-02	0.22	3.30	1.09	2.95E-01	2.95E-01	0.40
ATS1	0.54	4.62	6.23	1.26E-02	3.52E-02	0.04	4.62	0.04	8.38E-01	8.38E-01	0.08
LMO1	0.53	6.04	8.81	2.99E-03	1.09E-02	0.20	6.04	1.20	2.73E-01	2.73E-01	0.37
RFA1	0.53	7.41	7.24	7.14E-03	2.22E-02	0.40	7.41	4.17	4.12E-02	4.12E-02	0.76
IFH1	0.53	6.92	6.18	1.29E-02	3.58E-02	0.38	6.92	3.23	7.25E-02	7.25E-02	0.72
AAT21	0.53	7.27	15.31	9.12E-05	6.09E-04	0.35	7.27	6.64	9.96E-03	9.96E-03	0.66
UAP1	0.53	5.87	6.31	1.20E-02	3.40E-02	0.31	5.87	2.14	1.43E-01	1.43E-01	0.58
PMT2	0.52	6.87	6.24	1.25E-02	3.50E-02	0.77	6.87	13.49	2.40E-04	2.40E-04	1.48
SAP1	0.52	2.70	6.94	8.45E-03	2.57E-02	0.35	2.70	3.13	7.68E-02	7.68E-02	0.67
GPI13	0.51	4.14	6.93	8.49E-03	2.58E-02	0.30	4.14	2.34	1.26E-01	1.26E-01	0.58
PHM5	0.51	6.33	11.25	7.94E-04	3.63E-03	0.33	6.33	4.81	2.83E-02	2.83E-02	0.65
MYO2	0.51	6.18	7.37	6.63E-03	2.09E-02	0.43	6.18	5.19	2.27E-02	2.27E-02	0.84
MRS4	0.50	5.98	6.99	8.18E-03	2.50E-02	0.07	5.98	0.14	7.12E-01	7.12E-01	0.14
FHL1	0.50	5.73	8.30	3.97E-03	1.38E-02	0.05	5.73	0.07	7.89E-01	7.89E-01	0.09
PEX6	0.50	4.58	10.22	1.39E-03	5.80E-03	0.28	4.58	3.26	7.09E-02	7.09E-02	0.57
DAL81	0.49	5.43	5.83	1.57E-02	4.20E-02	0.13	5.43	0.41	5.23E-01	5.23E-01	0.26
KEX2	0.49	6.61	10.01	1.56E-03	6.39E-03	0.37	6.61	5.81	1.60E-02	1.60E-02	0.76
CTA8	0.49	6.88	6.40	1.14E-02	3.27E-02	0.33	6.88	2.81	9.39E-02	9.39E-02	0.66
CHS7	0.49	6.38	10.11	1.48E-03	6.12E-03	0.24	6.38	2.49	1.15E-01	1.15E-01	0.50

FGR10	0.48	2.95	9.25	2.35E-03	9.01E-03	0.36	2.95	4.94	2.63E-02	2.63E-02	**0.74**
BUD5	0.48	5.50	7.17	7.41E-03	2.29E-02	0.18	5.50	1.02	3.13E-01	3.13E-01	**0.38**
CGT1	0.48	5.86	17.34	3.12E-05	2.48E-04	0.21	5.86	3.19	7.42E-02	7.42E-02	**0.43**
MUB1	0.48	5.92	15.73	7.30E-05	5.00E-04	0.38	5.92	9.66	1.89E-03	1.89E-03	**0.78**
ARO80	0.48	4.88	8.98	2.73E-03	1.02E-02	0.47	4.88	8.55	3.45E-03	3.45E-03	**0.98**
DFR1	0.47	4.17	15.95	6.50E-05	4.54E-04	0.39	4.17	10.52	1.18E-03	1.18E-03	**0.82**
MP65	0.47	10.57	7.83	5.13E-03	1.71E-02	0.22	10.57	1.71	1.91E-01	1.91E-01	**0.47**
ALG8	0.46	4.21	9.69	1.85E-03	7.39E-03	0.28	4.21	3.61	5.75E-02	5.75E-02	**0.61**
MSC7	0.46	6.51	6.68	9.76E-03	2.90E-02	0.25	6.51	1.96	1.62E-01	1.62E-01	**0.54**
MNT4	0.46	3.00	7.42	6.44E-03	2.04E-02	0.52	3.00	9.33	2.25E-03	2.25E-03	**1.12**
RAD53	0.46	4.63	6.26	1.23E-02	3.46E-02	0.48	4.63	6.72	9.52E-03	9.52E-03	**1.04**
FEN12	0.45	5.73	8.78	3.05E-03	1.11E-02	0.31	5.73	4.23	3.97E-02	3.97E-02	**0.69**
PMT4	0.45	7.37	5.72	1.67E-02	4.41E-02	0.83	7.37	18.84	1.42E-05	1.42E-05	**1.82**
RCK2	0.45	7.86	17.96	2.25E-05	1.88E-04	0.10	7.86	0.87	3.50E-01	3.50E-01	**0.22**
PGA53	0.45	8.18	6.59	1.03E-02	3.01E-02	0.25	8.18	1.98	1.60E-01	1.60E-01	**0.55**
RAC1	0.45	6.03	9.42	2.15E-03	8.36E-03	0.15	6.03	1.09	2.98E-01	2.98E-01	**0.34**
AXL2	0.44	5.90	6.27	1.23E-02	3.45E-02	0.36	5.90	4.24	3.94E-02	3.94E-02	**0.82**
TRY2	0.44	5.17	9.86	1.68E-03	6.84E-03	0.07	5.17	0.25	6.19E-01	6.19E-01	**0.16**
SPE1	0.44	7.73	6.15	1.32E-02	3.63E-02	0.03	7.73	0.02	8.86E-01	8.86E-01	**0.06**
ABZ1	0.44	5.89	7.45	6.34E-03	2.02E-02	0.15	5.89	0.86	3.54E-01	3.54E-01	**0.34**
FOL1	0.44	4.72	8.63	3.31E-03	1.19E-02	0.38	4.72	6.54	1.06E-02	1.06E-02	**0.87**
CSO99	0.44	5.75	5.45	1.95E-02	4.96E-02	0.12	5.75	0.44	5.06E-01	5.06E-01	**0.28**
FLC1	0.44	7.00	6.20	1.28E-02	3.56E-02	0.06	7.00	0.13	7.17E-01	7.17E-01	**0.15**
PMR1	0.44	6.37	13.54	2.33E-04	1.32E-03	0.39	6.37	11.06	8.81E-04	8.81E-04	**0.90**
FAA4	0.43	8.99	9.88	1.67E-03	6.81E-03	0.07	8.99	0.28	5.96E-01	5.96E-01	**0.17**
APM1	0.42	6.20	9.15	2.49E-03	9.42E-03	0.31	6.20	5.15	2.33E-02	2.33E-02	**0.75**
TIP1	0.41	5.12	11.51	6.93E-04	3.25E-03	0.38	5.12	9.75	1.79E-03	1.79E-03	**0.92**
CHT1	0.41	4.15	6.57	1.03E-02	3.02E-02	0.00	4.15	0.00	9.81E-01	9.81E-01	**0.01**
RAD23	0.41	7.40	14.41	1.47E-04	8.86E-04	0.11	7.40	1.02	3.11E-01	3.11E-01	**0.27**
GDS1	0.39	8.37	6.18	1.29E-02	3.58E-02	0.53	8.37	11.14	8.45E-04	8.45E-04	**1.34**
DOT1	0.39	6.02	8.17	4.26E-03	1.47E-02	0.38	6.02	7.64	5.71E-03	5.71E-03	**0.97**
RAD7	0.38	5.08	5.68	1.72E-02	4.50E-02	0.49	5.08	9.18	2.44E-03	2.44E-03	**1.27**
CDC43	0.38	2.20	5.65	1.75E-02	4.57E-02	0.60	2.20	13.90	1.92E-04	1.92E-04	**1.58**
TAC1	0.38	5.32	5.92	1.50E-02	4.04E-02	0.15	5.32	0.98	3.23E-01	3.23E-01	**0.41**
SOH1	0.38	3.44	6.36	1.17E-02	3.33E-02	0.13	3.44	0.71	3.99E-01	3.99E-01	**0.33**
STT3	0.38	6.15	5.70	1.69E-02	4.46E-02	0.65	6.15	16.71	4.37E-05	4.37E-05	**1.72**
NOT5	0.37	6.54	8.81	3.00E-03	1.10E-02	0.02	6.54	0.02	8.90E-01	8.90E-01	**0.05**
RTG3	0.36	6.43	6.57	1.04E-02	3.03E-02	0.13	6.43	0.93	3.36E-01	3.36E-01	**0.38**
NGG1	0.35	5.83	11.48	7.04E-04	3.29E-03	0.21	5.83	3.93	4.73E-02	4.73E-02	**0.59**
LHS1	0.35	6.63	8.44	3.66E-03	1.29E-02	0.44	6.63	13.33	2.61E-04	2.61E-04	**1.26**
GVP36	0.35	6.05	6.86	8.81E-03	2.66E-02	0.36	6.05	7.47	6.27E-03	6.27E-03	**1.04**
ERG27	0.34	5.18	13.33	2.61E-04	1.44E-03	0.38	5.18	16.59	4.64E-05	4.64E-05	**1.12**
HST3	0.33	5.21	7.51	6.15E-03	1.97E-02	0.14	5.21	1.28	2.58E-01	2.58E-01	**0.41**
PEX22	0.32	5.36	5.64	1.75E-02	4.57E-02	0.21	5.36	2.45	1.17E-01	1.17E-01	**0.66**
RAD50	0.32	4.90	5.51	1.89E-02	4.83E-02	0.67	4.90	23.57	1.21E-06	1.21E-06	**2.08**
YBP1	0.31	6.03	6.33	1.19E-02	3.38E-02	0.45	6.03	12.91	3.28E-04	3.28E-04	**1.43**
PEX1	0.31	5.45	6.15	1.31E-02	3.62E-02	0.21	5.45	2.79	9.50E-02	9.50E-02	**0.67**
MEC1	0.30	5.28	6.03	1.40E-02	3.84E-02	0.53	5.28	18.59	1.62E-05	1.62E-05	**1.76**
OCH1	0.30	5.98	10.58	1.14E-03	4.97E-03	0.26	5.98	8.05	4.55E-03	4.55E-03	**0.87**
POM152	0.30	5.80	8.90	2.85E-03	1.05E-02	0.38	5.80	14.66	1.29E-04	1.29E-04	**1.28**
YAE1	0.28	4.62	5.92	1.50E-02	4.05E-02	0.10	4.62	0.81	3.67E-01	3.67E-01	**0.37**
VTC3	0.28	7.10	5.56	1.84E-02	4.73E-02	0.18	7.10	2.28	1.31E-01	1.31E-01	**0.64**
ECM39	0.28	5.68	6.27	1.23E-02	3.46E-02	0.29	5.68	7.05	7.94E-03	7.94E-03	**1.06**
SPP1	0.27	4.91	7.01	8.12E-03	2.48E-02	0.35	4.91	11.76	6.05E-04	6.05E-04	**1.30**
CKA1	0.27	6.57	6.02	1.42E-02	3.87E-02	0.07	6.57	0.40	5.26E-01	5.26E-01	**0.26**
RPB7	0.23	5.28	5.66	1.74E-02	4.53E-02	0.03	5.28	0.12	7.27E-01	7.27E-01	**0.15**
NUP85	0.23	6.25	7.08	7.81E-03	2.41E-02	0.28	6.25	10.67	1.09E-03	1.09E-03	**1.23**

LACTATE: UP-REGULATED GENES

>2-fold UP

>4-fold UP

Targets

Gene	RESPONDERS logFC	logCPM	LR	PValue	FDR	NON-RESPONDERS logFC	logCPM	LR	PValue	FDR	NON-R logFC / RESP log FC
MRV4	3.91	-0.93	15.02	1.06E-04	1.33E-02	2.82	-0.93	8.43	3.68E-03	3.41E-01	0.72
OSM2	3.55	6.64	73.83	8.50E-18	2.51E-14	2.70	6.64	45.89	1.25E-11	2.46E-08	0.76
HGT1	3.40	5.28	27.73	1.40E-07	5.89E-05	3.32	5.28	26.58	2.52E-07	2.48E-04	0.98
PGA31	3.39	2.13	23.62	1.17E-06	4.02E-04	0.21	2.13	0.11	7.40E-01	9.98E-01	0.06
TRY4	3.38	-0.44	20.48	6.03E-06	1.32E-03	0.64	-0.44	0.84	3.59E-01	9.98E-01	0.19
PRN3	3.21	5.65	31.69	1.81E-08	1.33E-05	0.28	5.65	0.29	5.93E-01	9.98E-01	0.09
FET31	2.87	6.23	51.08	8.85E-13	1.74E-09	2.81	6.23	48.91	2.68E-12	7.91E-09	0.98
HGT2	2.81	5.98	22.00	2.73E-06	6.71E-04	1.83	5.98	10.09	1.49E-03	2.25E-01	0.65
SOD3	2.77	4.67	20.91	4.80E-06	1.09E-03	2.59	4.67	18.51	1.69E-05	1.11E-02	0.93
CYB2	2.58	6.82	46.94	7.33E-12	1.08E-08	2.04	6.82	30.58	3.20E-08	3.77E-05	0.79
PRA1	2.44	-1.00	20.23	6.86E-06	1.39E-03	0.87	-1.00	2.51	1.13E-01	9.28E-01	0.35
RDN5	2.33	3.04	14.17	1.67E-04	1.79E-02	2.65	3.04	17.96	2.25E-05	1.21E-02	1.14
PGA26	2.10	2.33	13.35	2.58E-04	2.49E-02	0.70	2.33	1.56	2.11E-01	9.98E-01	0.33
HGT17	2.07	0.70	14.59	1.34E-04	1.49E-02	0.30	0.70	0.32	5.71E-01	9.98E-01	0.14
SFC1	1.83	4.53	13.19	2.81E-04	2.67E-02	0.89	4.53	3.28	7.02E-02	8.36E-01	0.49
ECM3	1.82	6.24	94.85	2.06E-22	1.21E-18	1.39	6.24	56.69	5.10E-14	3.01E-10	0.76
CFL2	1.79	5.51	13.00	3.12E-04	2.83E-02	1.91	5.51	14.56	1.36E-04	4.21E-02	1.06
PHHB	1.58	5.42	30.34	3.63E-08	1.95E-05	1.26	5.42	19.56	9.75E-06	7.19E-03	0.80
ENA2	1.51	6.02	19.97	7.85E-06	1.49E-03	1.08	6.02	10.56	1.16E-03	2.01E-01	0.72
ARD	1.34	9.17	20.34	6.49E-06	1.37E-03	0.68	9.17	5.33	2.09E-02	6.68E-01	0.51
CAT2	1.26	5.94	30.69	3.03E-08	1.79E-05	0.54	5.94	5.69	1.71E-02	6.44E-01	0.43
PRN1	1.15	3.11	15.20	9.69E-05	1.27E-02	0.83	3.11	8.07	4.50E-03	3.60E-01	0.72
GCV1	1.03	6.44	14.76	1.22E-04	1.47E-02	0.54	6.44	4.16	4.13E-02	7.60E-01	0.53
FOX2	1.01	5.59	13.87	1.96E-04	2.00E-02	0.44	5.59	2.71	9.99E-02	9.10E-01	0.44
DIP5	1.00	7.18	18.13	2.06E-05	3.29E-03	0.65	7.18	7.76	5.34E-03	3.98E-01	0.65
WAR1	0.96	6.00	11.71	6.22E-04	4.55E-02	0.51	6.00	3.33	6.80E-02	8.36E-01	0.53
PXA2	0.83	5.07	14.70	1.26E-04	1.47E-02	0.42	5.07	3.87	4.92E-02	7.97E-01	0.51
PXA1	0.80	5.93	15.60	7.84E-05	1.10E-02	0.35	5.93	3.07	7.99E-02	8.63E-01	0.44
ANT1	0.78	2.81	11.70	6.24E-04	4.55E-02	0.30	2.81	1.69	1.94E-01	9.96E-01	0.38
FOX3	0.76	4.72	12.69	3.67E-04	3.14E-02	0.57	4.72	7.13	7.59E-03	4.76E-01	0.75
MIS11	0.73	9.00	12.08	5.10E-04	4.11E-02	0.40	9.00	3.58	5.86E-02	8.22E-01	0.54
ECI1	0.71	5.21	12.19	4.81E-04	4.00E-02	0.61	5.21	9.04	2.64E-03	2.84E-01	0.86
STD1	0.66	6.93	22.68	1.91E-06	5.37E-04	0.23	6.93	2.85	9.15E-02	8.85E-01	0.35
PEX6	0.65	4.58	17.30	3.19E-05	4.95E-03	0.31	4.58	4.05	4.41E-02	7.62E-01	0.48
FAA2-3	0.56	3.25	13.57	2.30E-04	2.26E-02	0.15	3.25	0.99	3.20E-01	9.98E-01	0.27
PEX19	0.35	6.68	12.08	5.09E-04	4.11E-02	0.24	6.68	5.89	1.52E-02	6.28E-01	0.70
