# Supplementary material for: A CO2 sensing module modulates β-1,3-glucan exposure in Candida albicans
Source: mBio. 2024 Jan 23;15(2):e01898-23. doi: 10.1128/mbio.01898-23 (PMC10865862; doi:10.1128/mbio.01898-23)
Supplement: Table S6 — Stress and drug resistance ofC. albicans mutants. [file mbio.01898-23-s0008.pdf]

Supplementary Table 6. Stress and drug resistance of C. albicans mutants.

CONDITION / STRAIN	isolate	30°C YPD	37°C YPD	42°C YPD	45°C YPD	CO_2 YPD	37°C + FCS 5% YPD	NaCl 1M YPD
try6	A	2	2	2	2	2	2	2
	B	2	2	2	2	2	2	2
pho84	A	2	2	2	2	2	2	2
	B	2	2	2	2	2	2	2
xog1	A	2	2	2	2	2	2	2
	B	2	2	2	2	2	2	2
ctn1	A	2	2	2	2	2	2	2
	B	2	2	2	2	2	2	2
cyb2	A	2	2	2	2	2	2	2
	B	2	2	2	2	2	2	2
osm1	A	2	2	2	2	2	2	2
	B	2	2	2	2	2	2	2
ecm3	A	2	2	2	2	2	2	2
	B	2	2	2	2	2	2	2
hcm1	A	1	1	1	0	1	1	1
	B	1	1	1	0	1	1	1
try4	A	2	2	2	2	2	2	2
	B	2	2	2	2	2	2	2
nce103	A	0	ND	ND	ND	2	ND	2
	B	0	ND	ND	ND	2	ND	2

0 = No growth
1 = Less growth than wt
2 = Same growth as wt
3 = More growth than wt
ND = Not done

Notes:

A. Wild type showed no growth under this condition

KCl 0.6M YPD	H_2O_2 5mM YPD	Menadione 0.1mM YPD	Menadione 0.3 mM YPD	DTT 25 mM YPD	BPS 350 µM YPD	$FeCl_3$ 100 µM YPD	$CuSO_4$ 5 mM YPD
2	2	2	0	2	2	2	2
2	2	2	0	2	2	2	2
2	2	2	0	2	2	2	2
2	2	2	0	2	2	2	2
2	2	2	0	2	2	2	2
2	2	2	0	2	2	2	2
2	2	2	0	2	2	2	2
2	2	2	0	2	2	2	2
2	2	2	0	2	2	2	2
2	2	2	0	2	2	2	2
2	2	2	0	2	2	2	2
2	2	2	0	2	2	2	2
2	2	2	0	2	2	2	2
2	2	2	0	2	2	2	2
1	1	0	0	2	2	1	1
1	1	0	0	2	2	1	1
2	2	2	0	2	2	2	2
2	2	2	0	2	2	2	2
2	2	2	0	2	0	2	2
2	2	2	0	2	0	2	2

3-Aminotriazole 10 mM YPD	Acetic Acid pH3 20 mM YPD	Acetic Acid pH 6 20 mM YPD	pH 4.5 YPD	pH 7 YPD	pH 9.5 YPD	Glucose YNB
2	1	2	2	2	2	2
2	1	2	2	2	2	2
2	2	2	2	2	2	2
2	2	2	2	2	2	2
2	1	2	2	2	2	2
2	1	2	2	2	2	2
2	3	2	2	2	2	2
2	3	2	2	2	2	2
2	3	2	2	2	2	2
2	3	2	2	2	2	2
2	3	2	2	2	2	2
2	3	2	2	2	2	2
2	2	2	2	2	2	2
2	2	2	2	2	2	2
1	0	1	1	2	2	1
1	0	1	1	2	2	1
2	2	2	2	2	2	2
2	2	2	2	2	2	2
2	1	2	2	2	2	1
2	1	2	2	2	2	1

Lactate	Glyc&EtOH	Congo red	Calcofluor White	Caffeine	Fluconazole (µg	
		0.3 mg/ml	0.06 mg/ml	1 mg/ml	0.065	0.65
YNB	YNB	YPD	YPD	YPD	YPD	YPD
2	2	2	2	2	2	2
2	2	2	2	2	2	2
2	2	2	2	2	2	2
2	2	2	2	2	2	2
2	2	2	2	2	2	2
2	2	2	2	2	2	2
0	0	2	2	2	2	2
0	0	2	2	2	2	2
2	2	2	2	2	2	2
2	2	2	2	2	2	2
2	2	2	2	2	2	2
2	2	2	2	2	2	2
2	2	2	2	2	2	2
2	2	2	2	2	2	2
1	1	1	2	1	1	1
1	1	1	2	1	1	1
2	2	2	2	2	2	2
2	2	2	2	2	2	2
2	2	2	2	2	2	3
2	2	2	2	2	2	3

:/ml)	Caspofungin (µg/ml)			Ambisome [A]
6.5	0.032	0.32	3.2	5 µg/ml
YPD	YPD	YPD	YPD	YPD
0	2	2	2	2
0	2	2	2	2
0	2	2	2	2
0	2	2	2	2
0	2	2	2	2
0	2	2	2	2
0	2	2	2	2
0	2	2	2	2
0	2	2	2	2
0	2	2	2	2
0	2	2	2	2
0	2	2	2	2
0	2	2	2	2
0	2	2	2	2
0	1	1	1	2
0	1	1	1	2
0	2	2	2	2
0	2	2	2	2
0	2	2	2	2
0	2	2	2	2

Supplementary Table S6. Stress and drug resistance of *C. albicans* mutants.

Resistance to stressors and antifungal drugs was tested by plating serial dilutions of the pairs of independent homozygous null mutants (A,B) for the nine target loci on rich (YPD) or minimal media (YNB) containing the specified stressor at the specified concentration (e.g. Supplementary Figure 2). Plates were incubated at 30 °C (unless specified otherwise) for 48 h, imaged, and the growth in the presence of stressor was estimated relative to growth in the absence of stressor: 0, no growth, dark pink; 1, low growth, light pink; 2, normal growth, white; 3, enhanced growth, green. The data represent median values from three independent experiments. Note that *nce103* phenotypes were tested under 5% CO_2; nd, not done; na, not applicable.
