# Supplementary material for: A CO2 sensing module modulates β-1,3-glucan exposure in Candida albicans
Source: mBio. 2024 Jan 23;15(2):e01898-23. doi: 10.1128/mbio.01898-23 (PMC10865862; doi:10.1128/mbio.01898-23)
Supplement: Table S7 — β-1,3-Glucan exposure by C. albicans deletion mutants. [file mbio.01898-23-s0009.pdf]

Supplementary Table 7. β-1,3-glucan exposure by *C. albicans* deletion mutants.

	Glucose		Glucose + Lactate		Fold
Strain	Ave MFI	SD	Ave MFI	SD	Change
WT (SC5314)	4638	85	2605	187	0.56
ctn1 A	4432	405	2710	183	0.61
ctn1 B	4190	529	2911	266	0.69
cyb2 A	4183	333	2430	98	0.58
cyb2 B	4569	164	2515	132	0.55
ecm3 A	5109	464	2583	287	0.51
ecm3 B	5316	58	2913	102	0.55
hcm1 A	5139	671	2300	234	0.45
hcm1 B	5496	416	2223	179	0.40
nce103 A	3090	359	2622	222	0.85
nce103 B	2826	256	2474	147	0.88
osm1 A	5139	671	2115	57	0.41
osm1 B	5496	416	2199	99	0.40
pho84 A	3682	456	2875	79	0.78
pho84 B	3956	485	2814	34	0.71
try4 A	6099	914	3328	858	0.55
try4 B	5937	880	3442	117	0.58
try6 A	5880	1495	3248	185	0.55
try6 B	6973	742	3268	346	0.47

Supplementary Table S7. β-1,3-glucan exposure by *C. albicans* deletion mutants.

Wild type (SC5314) and mutant *C. albicans* cells (Supplementary Table 2) were grown in GYNB or GYNB plus lactate at 30 °C and their levels of β-1,3-glucan exposure quantified by Fc-dectin-1 staining and flow cytometry. Means and standard deviations from three independent replicate experiments are shown.
