# Supplementary material for: A CO2 sensing module modulates β-1,3-glucan exposure in Candida albicans
Source: mBio. 2024 Jan 23;15(2):e01898-23. doi: 10.1128/mbio.01898-23 (PMC10865862; doi:10.1128/mbio.01898-23)
Supplement: Table S8 — Oligonucleotide primers used in this study. [file mbio.01898-23-s0010.pdf]

Supplementary Table 8. Oligonucleotide primers used in this study

Oligo sequence obtained from Min et al (2016) https://msphere.asm.org/content/1/3/e00130-16/figures-only

Primer	Sequence (5' -> 3')	
CaCas9/for	ATCTCATTAGATTTGGAACTTGTGGGTT	Forward primer for amplification of *CaCas9* cassette
CaCas9/rev	TTCGAGCGTCCCAAAACCTTCT	Reverse primer for amplification of *CaCas9* cassette
SNR52/F	AAGAAAGAAAGAAAACCAGGAGTGAA	Forward primer for amplification of *SNR52* promoter
sgRNA/R	ACAAATATTTAAACTCGGGACCTGG	Reverse primer for amplification of sgRNA scaffold
SNR52/N	GCGGCCGCAAGTGATTAGACT	Forward and reverse nested primers for third round PCR for construction of
sgRNA/N	GCAGCTCAGTGATTAAGAGTAAAGATGG	sgRNA expression cassette

Reverse primer for amplification of *SNR52* promoter with overlapping guide sequence of the target gene

Guide Sequence in red

SNR52-sg-ECM3-Rv	CAGTGAAGGGTCAGCTGTAACAAATTAAAAATAGTTTACGCAAGTC
SNR52-sg-OSM2-Rv	CAGTCAAATCCAAATCGAAACAAATTAAAAATAGTTTACGCAAGTC
SNR52-sg-CYB2-Rv	CTACAAATAATGGCACCACACAAATTAAAAATAGTTTACGCAAGTC
SNR52-sg-NCE103-Rv	CCACAATCAGTATGACCACACAAATTAAAAATAGTTTACGCAAGTC
SNR52-sg-HCM1-Rv	CCCAACATCTCCCTTCTTATCAAATTAAAAATAGTTTACGCAAGTC
SNR52-sg-TRY4-Rv	AGGAAGTGAAGTTGCCGTCACAAATTAAAAATAGTTTACGCAAGTC
gma.SNR52.TRY6/R	AAATGATAACATACCTAGAACAAATTAAAAATAGTTTACGCAAGTC
gma.SNR52.CTN1/R	CGAGATATGCATTACAAGTTCAAATTAAAAATAGTTTACGCAAGTC
gma.SNR52.PHO84/R	AAATGAGAACAGAAATCTTTCAAATTAAAAATAGTTTACGCAAGTC

Forward primer for amplification of sgRNA scaffold with overlapping guide sequence of the target gene

Guide Sequence in red

ECM3-sg-scaf-Fw	TTACAGCTGACCCTTCACTGGTTTTAGAGCTAGAAATAGCAAGTTAAA
OSM2-sg-scaf-Fw	TTTCGATTTGGATTTGACTGGTTTTAGAGCTAGAAATAGCAAGTTAAA
CYB2-sg-scaf-Fw	TGTGGTGCCATTATTTGTAGGTTTTAGAGCTAGAAATAGCAAGTTAAA
NCE103-sg-scaf-Fw	TGTGGTCATACTGATTGTGGGTTTTAGAGCTAGAAATAGCAAGTTAAA
HCM1-sg-scaf-Fw	ATAAGAAGGGAGATGTTGGGGTTTTAGAGCTAGAAATAGCAAGTTAAA
TRY4-sg-scaf-Fw	TGACGGCAACTTCACTTCCTGTTTTAGAGCTAGAAATAGCAAGTTAAA
gma.sgRNA.TRY6/F	TTCTAGGTATGTTATCATTTGTTTTAGAGCTAGAAATAGCAAGTTAAA
gma.sgRNA.CTN1/F	AACTTGTAATGCATATCTCGGTTTTAGAGCTAGAAATAGCAAGTTAAA
gma.sgRNA.PHO84/F	AAAGATTTCTGTTCTCATTTGTTTTAGAGCTAGAAATAGCAAGTTAAA

Primers to amplify the repair template

NAT-ECM3-RT-Fw	GAAGAATTCTAGAAAACTAGAAATAATAGAAAGAATACCCACTTTAACTTTCACAAACTTTTATTGATTTCTGCAAAACCAGTCTAATCACTTGCGGCCGC
NAT-ECM3-RT-Rv	TGTTCACATAATGTATTATCCGTGTATATTAATTGTCTATAATGTTGAATCATCATCCTATAATTTGTGAATTATTATGTGGACCACCTTTGATTGTAAATAG
NAT-OSM2-RT-Fw2	TTTCATTAACTACAATCTAATACAATTATATATACATATTCCAATTGAGTCTTATAAAACTAAATTAACACTTTTCAACAAGTCTAATCACTTGCGGCCGC
NAT-OSM2-RT-Rv2	GCCATTAAATAAGTTATATGTCATTTTTTAAAAAAATAATAATGTATATCTAGTTTTTTTTTACACACTAACTAAATACGGACCACCTTTGATTGTAAATAG
NAT-CYB2-RT-Fw	CCCCAATTGATAAAAAGCTAACTCTCTATAGTTATTGTTGATTTCTACTACTACTACTACTATTACATCCTTTATAAAGTCTAATCACTTGCGGCCGC
NAT-CYB2-RT-Rv2	ACACCCCTTTTTCCTTTCTAATTGTTTGTAAACTGTAGTCTATATTATAATATTTAACTTCCTTATTTTTATATACTGTTGGACCACCTTTGATTGTAAATAG
NAT-NCE103-RT-Fw	CCAAAACTATTCTTGTACGACCAGTATCAGTGGCATCACCATCTCAATTGAGGAGAACGTTTTCAAAATTAAGTAAAAACAGTCTAATCACTTGCGGCCGC
NAT-NCE103-RT-Rv	AAATGACAACAATTATCTAAACCATACGAATATCACCAATATATTTATTATTATTATTATTATCTATAAACTCTATAAACGGACCACCTTTGATTGTAAATAG
NAT-HCM1-RT-Fw	TTCTACTTTATATTTAAGAGATAAACCAAAGACACTGAAACAACTAAGCGAAAAAAAAAATTAATATACCTTTGGATCAAGAGTCTAATCACTTGCGGCCGC
NAT-HCM1-RT-Rv	AAAAGTGATTTGCCCATTCCCAAAAAAACCAATACCCAGTAACCAAAAATACAAACACCATGGGCAACAAAAAGAGACACGGACCACCTTTGATTGTAAATAG
NAT-TRY4-RT-Fw	ACCCCATACTTAATATTAATTATTCATAACCTATTTATTCATTGATTATACAACTAAAAATTAAAATACCGAATTACACCAGTCTAATCACTTGCGGCCGC
NAT-TRY4-RT-Rv	CCCCATGAAAATATAAATCAATCACAAATCATATTAAGATTAAAAATAAATATTAGTTTTGCCAAAAGATTAAGTTATTGGGACCACCTTTGATTGTAAATAG
gma.NAT.TRY6.rep/F	TTTCTCAGGTAAACCATCTTTGTATTTGCCCATACCCATATATCTATAGACAGTATTGTTGTTCTTGCCACATATTAATTAGTCTAATCACTTGCGGCCGC
gma.NAT.TRY6.rep/R	AAAAGGAAATAGTAAAATCTAAAAATCGAAATGAAGTTGTCATTAAATGTGAATTCCCCAGGGGGTAAAACAAGAAATCCGGACCACCTTTGATTGTAAATAG
gma.NAT.CTN1.rep/F	TAATTGATTTAAGTACTGGTTTTACTTATTAGTTGAGACAACCTCCATATACATACATATATATATTTACTCCCTCGAAAAGTCTAATCACTTGCGGCCGC
gma.NAT.CTN1.rep/R	AAATAAATAAATAAATAAATAAATAAGCTTAACAAAATACATGATCATCATAAAAACTCAAAGAACAACAATTGTTCATTGGACCACCTTTGATTGTAAATAG
gma.NAT.PHO84.rep/F	CTCTTTTTGCTATATCACATTTACAACTAACACTATAGTTTTTACAAAAAGATGGTTGCTGAAATTCAARSCTCACACTAAGTCTAATCACTTGCGGCCGC
gma.NAT.PHO84.rep/R	TATAATAAAGTGAAAAAAAAAATATACAATTAAATTAAATTAAATTAAACAAAACAAAATCAAACAGATAGAAATGAAAAGGACCACCTTTGATTGTAAATAG

PCR Diagnostic Primers

ECM3-5V-Fw	CGCATTGATAGTGTATGC
ECM3-ORF5-Rv	GTCCCGACATGTTGAAACTG
ECM3-ORF3-Fw	GCATTCTACACAGACCCATTAAG
ECM3-3V-Rv	GTGTACAAATGGTTATGTTG
OSM2-5V-Fw	CTTCCCCCACACACTCATTC
OSM2-ORF5-Rv	GATAAAATTGTTCAACCGAATCAGGG
OSM2-ORF3-Fw	GGAAGAGATGCTACTGAAAGTTTTGC
OSM2-3V-Rv	GGGGTTAATCTCAAATTAG
CYB2-5V-Fw	GGAGTCATTTGATAACAAATG
CYB2-ORF5-Rv	GGTGCCATTATTTGTAGTGGC
CYB2-ORF3-Fw	GCCATGTCAGGATATGGAGATG
CYB2-3V-Rv	CTTACTACTCTATTTACTACTATGATCTAC
NCE103-5V-Fw	ATCCCTTCCATCGTGTTGAC
NCE103-ORF5-Rv	GGAAAATTATTATGATTTCCTGAACTAAC
NCE103-ORF3-Fw	GAAGAATGAAATTGAAGTTTGGGGG
NCE103-3V-Rv	GCGATGCAATTGTTATTGTTAAATC
HCM1-5V-Fw	AACTGTTCACTGCCGTTGC
HCM1-ORF5-Rv	GCTGATATACTTTCAGGTGGTGTG
HCM1-ORF3-Fw	GTATGACGATGACGATATGGTTTCAAG
HCM1-3V-Rv	GGCTGTGCTTCTAGTGATTAATC
TRY4-5V-Fw	CCTACCCACCCACTCATATTCC
TRY4-ORF5-Rv	TGTGGTGGTGCTTGAGGTAG
TRY4-ORF3-Fw	CTTGACTAATGGATCTGTACCGTC
TRY4-3V-Rv	CATTCTCCCTACAAAAAGGAGGG
PHO84-5V-Fw	GGGTTTCCTGCGTATATACATAC
PHO84-ORF5-Rv	CCATTCCAGTAAACGTATTGTAAC
PHO84-ORF3-Fw	CTACTGCTCATGGTTTATCTGC
PHO84-3V-Rv	GACATTCCCCATCACAAAATC
TRY6-5V-Fw	CATTCTATACTTATCACGCACG
TRY6-ORF5-Rv	GCCGGAAACCTCTATTATGG
TRY6-ORF3-Fw	GTGAATTCAGGTAGTGCAAG
TRY6-3V-Rv	CTATCTAAAGGGTGTCTTCAC
CTN1-5V-Fw	CTCCTTCAAATCTCCCGCCC
CTN1-ORF5-Rv	CAACGGGGGAATCATAATTC
CTN1-ORF3-Fw	GGTTATGGATATTTTGATATGGGCG
CTN1-3V-Rv	CTGTCTTTTTGCAGCCGTCAC
NAT-5V-Rv	tataaatagcacacacccac
NAT-3V-Fw	gtactggtactggttctcgg
